# Subspecies differentiation and range‐wide genetic structure are driven by climate in the California gnatcatcher, a flagship species for coastal sage scrub conservation

**DOI:** 10.1111/eva.13429

**Published:** 2022-06-29

**Authors:** Amy G. Vandergast, Barbara E. Kus, Dustin A. Wood, Elizabeth R. Milano, Kristine L. Preston

**Affiliations:** ^1^ U.S. Geological Survey Western Ecological Research Center San Diego California USA; ^2^ Present address: U.S. Forest Service Rocky Mountain Research Station, Maintaining Resilient Dryland Ecosystems Moscow Idaho USA

**Keywords:** demographic history, gene–environment associations, habitat fragmentation, *Polioptila californica*, RADseq, SNPs

## Abstract

Understanding genetic structure and diversity within species can uncover associations with environmental and geographic attributes that highlight adaptive potential and inform conservation and management. The California gnatcatcher, *Polioptila californica*, is a small songbird found in desert and coastal scrub habitats from the southern end of Baja California Sur to Ventura County, California. Lack of congruence among morphological subspecies hypotheses and lack of measurable genetic structure found in a few genetic markers led to questions about the validity of subspecies within *P. californica* and the listing status of the coastal California gnatcatcher, *P. c. californica*. As a U.S. federally threatened subspecies, *P. c. californica* is recognized as a flagship for coastal sage scrub conservation throughout southern California. We used restriction site‐associated DNA sequencing to develop a genomic dataset for the California gnatcatcher. We sampled throughout the species' range, examined genetic structure, gene–environment associations, and demographic history, and tested for concordance between genetic structure and morphological subspecies groups. Our data support two distinct genetic groups with evidence of restricted movement and gene flow near the U.S.‐ Mexico international border. We found that climate‐associated outlier loci were more strongly differentiated than climate neutral loci, suggesting that local climate adaptation may have helped to drive differentiation after Holocene range expansions. Patterns of habitat loss and fragmentation are also concordant with genetic substructure throughout the southern California portion of the range. Finally, our genetic data supported the morphologically defined *P. c. californica* as a distinct group, but there was little evidence of genetic differentiation among other previously hypothesized subspecies in Baja California. Our data suggest that retaining and restoring connectivity, and protecting populations, particularly at the northern range edge, could help preserve existing adaptive potential to allow for future range expansion and long‐term persistence of the California gnatcatcher.

## 1 INTRODUCTION

The evolutionary history and contemporary distribution of genetic variation across species ranges are important to understand and inform species conservation efforts. Genetic data can help to determine appropriate evolutionary and geographic boundaries for management (Bickford et al., 2007; Palsbøll et al., 2007; Waples & Gaggiotti, 2006). They provide information on factors affecting gene flow and effective population size changes over time to inform habitat restoration and augmentation (Hohenlohe et al., 2021; Holderegger & Di Giulio, 2010; Keyghobadi, 2007; Ralls et al., 2018). Additionally, surveys of genome‐wide variation can capture both neutral and adaptive variation, thus offering additional insight into adaptive potential as well as increasing power and accuracy in estimating genetic structure and demographic history in comparison to studies of fewer loci (Allendorf et al., 2010; Funk et al., 2019).

When examined alongside morphological and ecological data, genomic variation can help delineate taxonomic units, including distinct population segments, subspecies, and species (Coates et al., 2018). Subspecies remain critical units for conservation purposes despite considerable ongoing debate in the scientific literature about the criteria for defining them (reviewed in Haig et al., 2006). Under the U.S. Endangered Species Act of 1973, listable entities for vertebrates include species, subspecies and distinct population segments. In birds, for example, approximately 44% of U.S. federally listed taxa are listed as subspecies (Haig & D'Elia, 2010). A subspecies has been broadly defined as a breeding population or collection of populations that occupies a distinct segment of the geographic range of the species and that is measurably distinct in phenotype, genotype or a combination of these traits (Avise, 2004; Haig & D'Elia, 2010; Mayr, 1969; Patten, 2010; Remsen, 2010). Classically, North American avian subspecies were described based on variation in measurements and plumage among museum specimens (James, 2010). Genetic data have provided power to distinguish among morphologically similar taxa, and have been critically important in identifying previously cryptic subspecies and species in birds and other taxa (Bickford et al., 2007; Fleischer et al., 2006; Funk, Mullins, Forsman, & Haig, 2007; Klicka et al., 2016). However, often genetic data are not congruent with morphological subspecies (Ball & Avise, 1992; Draheim et al., 2010; Funk, Mullins, & Haig, 2007; Wood et al., 2008). Further, there is debate concerning quantifying minimum distinctiveness for conservation‐informative units, ranging from strict reciprocal monophyly (Moritz, 1994; Zink, 2004), to pairwise statistical support (McCormack & Maley, 2015; Patten, 2010), to evidence of adaptive distinctiveness (Crandall et al., 2000). In cases where relatively few and unknown genes code for phenotypic traits under selection and/or the time scale of differentiation is recent, discordance between morphological traits and neutral genetic markers may be expected (McCormack & Maley, 2015; Winker, 2010). For these reasons, it may be difficult to apply a standard set of criteria to define all subspecies (Fitzpatrick, 2010).

**FIGURE 1:**
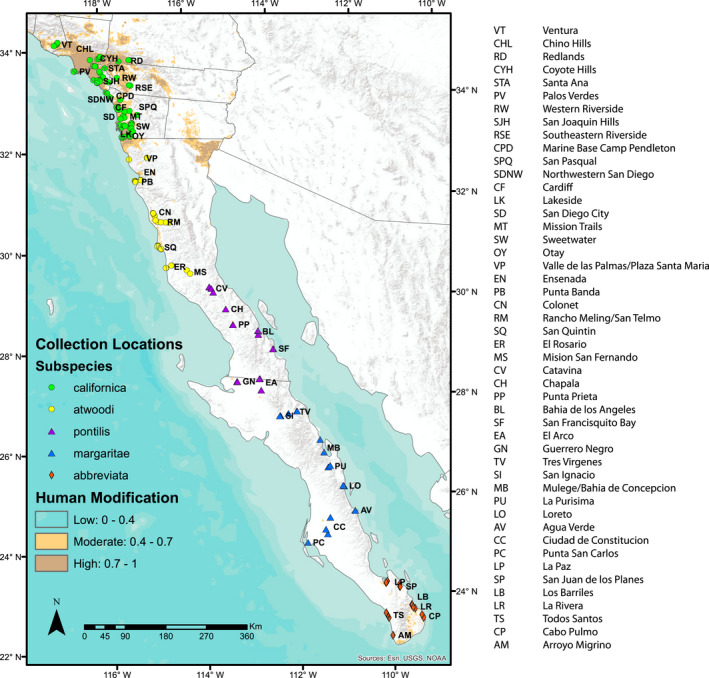
Sampling locations of 184 California gnatcatchers included in this study with locality names listed to the right. Colors correspond to Mellink and Rea (1994) subspecies groupings while symbol shapes correspond to Atwood (1991) subspecies groupings (circles = *californica*, triangles = *margaritae*, diamonds = *abbreviata*). Human modified habitat is shown in orange (moderate development) and brown (high development)

This debate is exemplified in the case of the California gnatcatcher, *Polioptila californica*. This small songbird is distributed in arid scrub and coastal sage scrub habitats from the southern tip of Mexico's Baja Peninsula north to Ventura County, California (Figure 1). Climate conditions vary dramatically across the species' range. At the northern end, southern California and northwestern Baja California experience cold and wet winters. Here, energetic constraints are hypothesized to limit the eastern and northern distribution of gnatcatchers (Mock, 1998), and cold wet winters are associated with increased mortality (Atwood et al., 1998; Erickson & Miner, 1998; Mock, 1998; Mock & Bolger, 1992; Preston et al., 1998). With the exception of high elevation mountains, the rest of the Baja Peninsula is dry and hot, especially the Vizcaíno Desert in the middle of the peninsula. In addition, the North American Monsoon consists of summer rains that are greatest at the southern end of the Baja Peninsula and extend northeast over the Gulf of California to Arizona (Cavazos & Arriaga‐Ramírez, 2012). Morphological variation across the range, particularly in plumage color and tail length, has been described and revised in an array of 3–5 subspecies (AOU, 1957; Atwood, 1991; Grinnell, 1926; Mellink & Rea, 1994; van Rossem, 1931a, 1931b). Due to habitat loss and population declines in the U.S. portion of its range, the subspecies *P. c. californica* was listed as threatened in 1993 under the U.S. Endangered Species Act (USFWS, 1993).

The subspecies taxonomy of California gnatcatcher has been the subject of revision and critique (Atwood, 1991; Cronin, 1997), which has, in turn, led to debate on the validity of the listing status of *P. c. californica*. Previous genetic studies of *P. californica* that examined a small number of nuclear and mitochondrial loci, and applied a criterion of reciprocal monophyly, concluded that there was no evidence of evolutionarily significant divisions among these, and therefore no valid subspecies (Zink et al., 2000, 2013). The conclusions of Zink et al. (2013) were, in turn, critiqued (McCormack & Maley, 2015; Patten, 2015). Specifically, McCormack and Maley (2015) suggested that reciprocal monophyly would be unlikely in cases with recent or ongoing gene flow among neighboring subspecies, and instead, suggested hypothesis testing for population distinctiveness among neighboring subspecies. Subsequently, a scientific panel convened by the U.S. Fish and Wildlife Service suggested that the available genetic data were not sufficient to overturn long‐standing conclusions based on phenotypic data and recommended gathering genome‐wide genetic data, testing for putatively adaptive loci, and collecting additional morphological data (AMEC, 2015).

To address these recommendations, we describe genomic variation across the range of the California gnatcatcher using a large dataset of single nucleotide polymorphisms (SNPS), and systematic sampling of the species' range. We applied clustering and landscape genetic methods to describe population structure and habitat and climate drivers of genetic differentiation. To assess putative adaptive responses to climate, we tested for genetic‐environment associations using climatic variables and examined the spatial structure in a subset of climate‐associated outlier loci. We explored the demographic history inferred from the genomic dataset, and estimated contemporary effective population size and genetic diversity across the range. Finally, we formally tested for concordance between genetic structure and the two most comprehensive morphological subspecies hypotheses of Mellink and Rea (1994) and Atwood (1991). Together, results provide a genomic framework that can inform conservation and management.

## 2 MATERIALS AND METHODS

### 2.1 Sample collections and DNA extraction

Our study area included the full species' range. Samples north of the international border (hereafter southern California) were collected between Spring 2012 and Fall 2013. Samples south of the international border were collected in Fall 2018 and Spring 2019, with locations chosen to equally represent the full species range throughout the Baja Peninsula (hereafter, Baja). We used established techniques to capture adults in mist nets strategically placed within territories, using song playbacks to attract birds to nets. Pin feathers were collected and placed in 500 μl of either Qiagen ATL Buffer (in southern California) or 1X DNA/RNA Shield Buffer (in Baja) and stored on ice until samples could be transferred to a −20° freezer. All birds were handled in accordance with protocols approved by the USGS Western Ecological Research Center Animal Care and Use Committee and permitted under U.S. Master Banding Permit 22372, U.S. Federal Permit 829554, and a Memorandum of Understanding with the California Department of Fish and Wildlife held by B. E. Kus, and Mexico collection permits of R. Rodriguez‐Estrella (Centro de Investigaciones Biológicas del Noroeste S.C.).

Feathers were extracted with the Gentra Puregene Kit (Qiagen) according to manufacturer's protocol with minor modifications, including cell lysis in the presence of Proteinase K and dithiothreitol (DTT), an overnight DNA precipitation step, and 5–30 min centrifugation steps at 21,194 *g* and 4°C. For samples with low yields, the same protocol was performed on the feather storage buffer. Feathers collected in Baja were extracted with the DNA Tissue Extraction Kit (Qiagen) according to manufacturer's protocol, with an extended 48 h digestion step in the presence of DTT. For samples with low yields, additional DNA was extracted from the storage buffer, using a standard salt precipitation method (Miller et al., 1988).

### 2.2 Library preparations and genomic data collection

In total 230 samples were prepared for sequencing at the Western Ecological Research Center, San Diego Field Station laboratory. We quantified DNA on a Qubit fluorometer (Life Technologies™), and 125–500 nanograms (ng) of DNA were used for next‐generation sequencing (NGS) library preparation. We followed the double‐digest restriction‐associated DNA (ddRAD) sequencing protocol developed in Peterson et al. (2012) for NGS library preparation modified to use separate indexing reads. We digested genomic DNAs using 20 units each of the restriction enzymes *EcoR*I and *Mse*I (New England Biolabs) and used Solid Phase Reversible Immobilization (SPRI) beads (Beckman Coulter) to purify the digestions prior to ligating uniquely bar‐coded adapters with T4 ligase (New England Biolabs). We quantified all ligation products on the Qubit fluorometer, pooled across index groups in equimolar concentrations, and then size selected fragments between 250 and 400 base pairs (bp) using a Pippin Prep size fractionator (Sage Science). We amplified the recovered fragments from each pool using 5–10 ng of the recovered DNA, Phusion High‐Fidelity *Taq* (New England Biolabs), and Illumina's primers (Illumina, Inc.). Polymerase chain reaction (PCR) products were then cleaned with SPRI beads and quantified using the Qubit fluorometer (Life Technologies) before being pooled for sequencing (100 bp single end reads) on two lanes of an Illumina HiSeq 4000 at the Genomics and Cell Characterization Core Facility at the University of Oregon. Samples from different sampling locations and regions were mixed across libraries and sequencing lanes to avoid confounding batch effects.

### 2.3 Bioinformatics

We filtered and selected datasets using the STACKS v. 2.53 (Catchen et al., 2013) bioinformatics pipeline. We removed reads with uncalled or low‐quality bases (phred score < 10) and discarded reads without any recognizable barcode adaptors using the *process_radtag* program within STACKS. Cleaned raw reads were aligned to the reference genome of the closest available relative, *Polioptila caerulea*, (NCBI accession GCA_013397295.1; estimated divergence time ~ 4 mya; Moura et al., 2018). Alignments were performed using BWA‐MEM aligner (http://bio‐bwa.sourceforge.net) and were then exported and sorted as .bam files using samtools (Li et al., 2009). The resulting alignment was then run through the *ref_map.pl* program within STACKS. All datasets were then generated using the *populations* module, retaining only loci present in at least 80 percent of individuals. The proportion of missing data per sample was calculated using a custom python script and samples missing more than 30 percent data were discarded. Two datasets were generated: a dataset with all loci retained, and one removing loci with a minor allele frequency (MAF) < 0.05. The latter dataset was used for range‐wide genomic structure and distance analyses, while the full dataset was used for demographic and gene–environment associations. Including low frequency alleles can increase the number of non‐synonymous mutations in the dataset and detection of true positive loci under selection (Ahrens et al., 2021).

### 2.4 Data analysis

Our analyses were structured to address four major objectives: (1) describing overall genetic structure and assessing relationships among genetic, habitat and climate distances; (2) testing for climate adaptation and identifying outlier loci; (3) exploring demographic history of the species and contemporary patterns of genetic diversity and effective population size; (4) testing for genetic distinctiveness in relation to two previous morphological subspecies hypotheses (Atwood, 1991; Mellink & Rea, 1994).

#### 2.4.1 Range‐wide genetic structure

We used two clustering methods to assess structure. First, we applied the maximum‐likelihood approach of ADMIXTURE (Alexander et al., 2009) to define the best supported number of genetic clusters (K) in the data. We performed 5 replicate analyses to evaluate up to 6 populations, to include the maximum number of morphological subspecies (5; Atwood, 1991; Mellink & Rea, 1994) plus one. To assess the best value of K, we performed 10‐fold cross‐validation and determined the K‐values with the lowest cross‐validation error and examined the individual assignment plots. We also used the software package fineRADstructure (Malinsky et al., 2018) to infer population structure using a model‐based Bayesian clustering approach that groups individuals together using levels of shared coancestry. We used the *RADpainter* module of fineRADstructure to create the input coancestry matrix, which is defined as a summary of nearest neighbor haplotype relationships. We used the *finestructure* module to assign individuals to populations using the default parameters of 100,000 Markov chain Monte Carlo (MCMC) iterations with a burn‐in of 100,000 iterations and sampling occurring every 1000 iterations. Finally, a tree was constructed with 10,000 hill‐climbing iterations, and a cladogram and heatmap were used to visualize the results using the provided R scripts (available at http://cichlid.gurdon.cam.ac.uk/fineRADstructure.html).

Next, we tested for associations between individual pairwise genetic distances and pairwise geographic, human modified habitat, and climate distances, as well as differences between the California and Baja groups supported in fineRADstructure and discriminant analyses (see section 2.4.4 for discriminant analysis methods). For distance‐based tests, we calculated Nei's genetic distances (Nei, 1972) among all pairs of individuals using StAMPP v. 1.6.2 in R (Pembleton et al., 2013). Euclidean distances and modified habitat cost distances among all individuals were calculated in ArcMap v. 10.4.1 using the Landscape Genetics Toolbox. Habitat loss in southern California and in northern Baja have been associated with reductions in occupancy and may impact connectivity (Atwood & Bontrager, 2020). We used the global human modification of terrestrial systems model, v. 1 (Kennedy et al., 2020) to estimate converted lands. This model provides a cumulative measure of human land modification at a 1‐km resolution. Following Kennedy et al. (2019), we assigned values below 0.4 as low to moderate modification, 0.4–0.7 as high modification, and >0.7 as very high modification. Costs of 1, 2 and 3 were assigned to each bin, respectively. We selected climate variables important in describing gnatcatcher habitat, survival, and range limitations, which could be important drivers of differentiation (Mock, 1998; Preston et al., 1998; Preston et al., 2020). Climate variables were downloaded from WordClim 2.1 Bioclimatic variables (1‐km^2^ resolution; 30‐year average 1970–2000; https://worldclim.org/data/worldclim21.html; accessed June 2021). We used maximum temperature of the warmest month (BIO5; hereafter TMax), minimum temperature of the coldest month (BIO6; hereafter TMin), precipitation seasonality (Coefficient of Variation; BIO15; hereafter PrecipS), precipitation of wettest quarter (BIO16; hereafter PrecipWQ), and precipitation of driest quarter (BIO17; hereafter PrecipDQ; see maps in Figure S1). Values were extracted for each collection point in ArcGIS v. 10.4.1, standardized, and Euclidean distance matrices for each were calculated in Primer v.7.0.13 (Clarke & Gorley, 2015). Finally, Mantel tests for matrix correlations and Multiple Matrices Regression (MMR) models were performed in the R package ecodist v. 2.0.7 (Goslee & Urban, 2007). MMR models were selected using stepwise elimination of variables that were positively correlated with genetic distance using Mantel tests. Significance of tests was assessed using 1000 permutations. Tests were run for the entire dataset and separately for California and Baja to determine whether different variables explained patterns of genetic differentiation in different parts of the range.

#### 2.4.2 Climate adaptation and genetic structure in putatively adaptive loci

Our second objective was to test for associations between climate variables and genetic variation. Because loci under selection may evolve at different rates than neutral genetic variation, we also explored whether patterns of genetic structure in climate‐adapted loci differed from the climate neutral dataset.

We used a redundancy analysis (RDA, van den Wollenberg, 1977) to determine how groups of loci covary in response to climate. RDA first uses multiple linear regression of response variables (genotypes) on predictor variables (climate data). Second, a matrix of the fitted values of all response values is subjected to PCA (Principal Components Analysis). RDA is a sensitive method for determining combinations of multiple loci that are associated with environmental variables (Forester et al., 2018). Analyses were performed in the R package vegan v2.5–6 (Oksanen et al., 2020) using individual genotype data and the 5 climate variables described above. We chose to perform this analysis at the level of individuals and not grouped by cluster or morphological subspecies for two reasons: first, the individual is the primary unit of selection (Lewontin, 1970); second, genetic clusters and subspecies cover very large geographic areas with differing climate conditions that would be obscured if collapsed into range means (see Figure S1). Genetic data were mean‐centered and missing data were imputed using the most common genotype at each SNP, following Forester et al. (2018). Climate variables with a variance inflation factor (VIF) <10 were retained. We mapped the RDA loadings (which represent linear combinations of predictor climate variables) of individual genotypes to examine spatial patterns in climate selection gradients. Points were interpolated across the sampled range for visualization purposes. We identified outlier loci greater than three standard deviations away from the mean RDA axes scores. To compare genetic structure in climate outlier SNPS versus the remainder of climate neutral SNPS, principal components analysis was performed in R using adegenet v. 2.1.3 (Jombart & Ahmed, 2011), and plots of the first and second PC axes were compared. The optimal number of clusters in each dataset was estimated using k‐means clustering and the Bayesian Information Criterion (BIC).

#### 2.4.3 Demographic history and contemporary Ne

Our third objective was to explore the demographic history quantifying the size and structure of past populations of the species. We used the diffusion approximation method implemented in the simulation software δaδi (Gutenkunst et al., 2009) to evaluate the likelihoods of alternative demographic models for *P. californica*. We used a one‐dimensional Joint Site Frequency Spectrum (JSFS), where the number of dimensions used in models refers to the species or distinct group, and a folded JSFS because we lacked out‐group information. Using the demographic modeling workflow from Portik et al. (2017), we fit six established models that broadly fall under one‐, two‐ and three‐epoch demographic histories involving population growth, population bottlenecks, or a null model of population stability.

Following the recommendations in the δaδi manual, we ‘projected down’ the dataset to a smaller sample size to account for missing data. We used the *dadi‐test‐projections.py* from Portik et al. (2017) to explore a range of sample sizes to maximize the total number of segregating sites and settled on a projection size of 296 alleles (80,125 segregating sites; projection size = number of individuals * 2 for diploid species). We ran five sets of increasingly focused optimizations for each model before performing the final model selection following the δaδi pipeline (Portik et al., 2017). For each round, we ran multiple replicates and used parameter estimates from the best scoring replicate to seed searches in the following round (replicates = 10, 20, 20, 50, 50; maxiters = 10, 20, 30, 50, 50; folds = 3, 2, 2, 2, 1), and optimized parameters using the Nelder–Mead method. Because not all models were nested under the same hierarchy, we ranked models by log likelihoods, the fit of the model‐data plots, and whether parameters converged to determine the best fitting model. To scale our transformed empirical values, we used a mutation rate (2.3 x 10 ^−9^ substitutions/site/generation) that was estimated for another small passerine (Smeds et al., 2016) and assumed a generation time of 1 year on the basis of published age structure and breeding activity records from two sites (Atwood & Bontrager, 2020). We also used δaδi to estimate the following summary statistics for the rangewide dataset and each grouping from our population structure analyses: Watterson's Theta (ϴ), Tajima's D, and the number of pairwise differences.

We estimated contemporary effective population size (N_e_) using the linkage disequilibrium method (Waples & Do, 2008) within the program NeEstimator v. 2.1 (Do et al., 2014). We assumed random mating for each group, calculated 95% confidence intervals for point estimates using the jackknife‐across‐samples method (Jones et al., 2016) and screened out rare alleles using a critical cut‐off value (*p*_*crit*_) of 0.05. We applied a standard correction for physical linkage based on the number of chromosomes as suggested by Waples et al. (2016) for genomic datasets. We used the average number of chromosomes reported for Certhioidae, the superfamily of wrens and their allies, 2 N = 76; 1 N = 38; Degrandi et al., 2020), as there were no reported chromosome counts for Polioptilidae.

#### 2.4.4 Tests for subspecies distinctiveness

Our final objective was to formally test whether our genomic data support previously hypothesized subspecies divisions in California gnatcatchers. We focused on Atwood's (1991) study of the entire species range and Mellink and Rea's (1994) study of northern Baja California. These studies examined large numbers of specimens lacking in earlier treatments (e.g. Grinnell, 1926; van Rossem, 1931a, 1931b). Atwood (1991) found differences in plumage coloration and tail length among three subspecies, with *P. c. californica* extending from the northern‐most edge south to 30°N latitude, *P. c. margaritae* in central Baja California to 24°N, and *P. c. abbreviata* in the Cape region of Baja California Sur, south of 24°N. Based on plumage color of newly collected specimens, Mellink and Rea (1994) revised the southern range extent of *P. c. californica* to the international border at approximately 32°N and described two additional subspecies: (*P. c. atwoodi*: 32°–30°N; *P. c. pontilis*: 30°–28°N) and *P. c. margaritae* south of 28°N, but did not comment on any breaks farther south. Following Zink et al. (2000), subspecies hypothesis 1 combined Mellink and Rea's (1994) latitudinal breaks with Atwood's break at the southern tip of the range, resulting in 5 subspecies (Table 1). Subspecies hypothesis 2 consisted of just Atwood's latitudinal breaks among 3 subspecies.

**TABLE 1:** California gnatcatcher morphological subspecies hypotheses, testing frameworks and support, with + designating presence of statistical support and NS non‐significance

Taxa and latitudinal divisions (N to S)	DAPC All Loci	Mantel Tests All Loci	AMOVA All Loci	AMOVA Climate Associated Loci
Hypothesis 1: Mellink and Rea (1994); Atwood (1991) below 24°N				
*californica* (S to 32°N)	+	+	+	+
*atwoodi* (32°–30°N)	NS	NS	NS	+
*pontilis* (30°–28°N)	NS	NS	NS	NS
*margaritae* (28°–24°N)	NS	NS	NS	NS
*abbreviata* (S of 24° N)	NS	NS	NS	NS
Hypothesis 2: Atwood (1991)				
*californica* (S to 30°N)	NS	NS	+	+
*margaritae* (30°–24°N)	NS	NS	NS	NS
*abbreviata* (S of 24°N)	NS	NS	NS	NS

We applied four testing frameworks. First, using all loci, we applied a discriminant analysis of principal components (DAPC), using either hypothesis 1 or hypothesis 2 as a priori groupings. To minimize overfitting, an initial DAPC was used to find the a‐score, which was used to select the number of principal components to retain in the subsequent re‐analysis (Jombart & Ahmed, 2011). Resulting posterior probabilities of assignments were examined to determine the proportion of sampled individuals in a subspecies range that could be assigned with high probability (>90%) to the correct subspecies group. We used a 75% cut‐off to assess whether a subspecies was diagnosable [e.g., assignment of >90% across 75% or more of the range (Patten & Unitt, 2002)]. Second, we tested for group differences in individual pairwise genetic distances for hypothesis 1 and 2 groupings. Finally, for hypothesis 1 and 2 groupings, we performed hierarchical Analysis of Molecular Variance (AMOVA) tests for significant differences in genetic variance among subspecies, among populations, and within populations, as previously suggested by McCormack and Maley (2015). These tests were performed in R using adegenet v. 2.1.3 (Jombart & Ahmed, 2011) and poppr v. 2.9.1 (Kamvar et al., 2014). AMOVAs with significant among‐subspecies (Ф_CT_) differences were followed with post‐hoc tests between group pairs. McCormack and Maley (2015) emphasized that the most informative pairwise tests should be those between geographically neighboring groups. Significance in all tests was assessed with 999 permutations, and the Benjamini and Yekutieli (2001) correction for multiple tests was applied, as recommended for genetic data comparisons (Narum, 2006). We performed AMOVAs using both the full genomic dataset and the subset of climate‐related outlier SNPs identified in the RDA.

## 3 RESULTS

### 3.1 Data quality

A total of 607.0 of 658.6 million reads (92.4%) were aligned to the reference genome with the remainder discarded due to insufficient mapping quality or excessive soft‐clipping. The average number of aligned reads per sample was 3.3 million. From these reads, a total of 724,435 loci were built and effective per‐sample coverage ranged from 5.0x to 57.2x, mean 18.1x (stdev, 11.1x). After quality filtering and dropping samples with read coverage lower than 8x and missing data greater than 30 percent, the final genomic dataset included 184 individuals from 48 aggregations across the California gnatcatcher range, with 84 individuals from the US and 100 from Baja California retained (Figure 1a; Table S1). The full dataset consisted of 84,125 loci with a total of 215,220 SNPs and 7.1% missing data. For demographic, genetic diversity and gene–environment analyses, we restricted the full dataset to only a single random SNP per RAD locus to avoid linkage disequilibrium (Andrews et al., 2016) for a total of 84,125 SNPs. For genetic structure and distance analyses, we further restricted the dataset by using the 0.05 minor allele frequency (MAF) cutoff, which resulted in 35,440 SNPs. Additional summary statistics are found in Table 2.

**TABLE 2:** Genome‐wide population summary statistics from stacks and δaδi outputs including number of individuals, private alleles, polymorphic sites, average observed heterozygosity (Hobs), and average nucleotide diversity (pi); summary statistics using the site frequency spectrum (JSFS) includes projection size, Waterson's Theta, Tajima's D, and average number of pairwise differences

Dataset	Statistic	so. CA	Baja	Rangewide
Full dataset	Number of individuals	84	100	184
	Private Alleles	28,673	74,980	NA
	Polymorphic sites	140,240	186,547	215,220
	Hobs	0.074	0.079	0.076
	pi	0.0021	0.0020	0.0021
JSFS from δaδi	Projection size	146	152	296
	Watterson's Theta	9326	11,073	12,788
	Tajima's D	−1.094	−1.610	−1.590
	Pairwise differences	6245	5675	6335

### 3.2 Population structure

**FIGURE 2:**
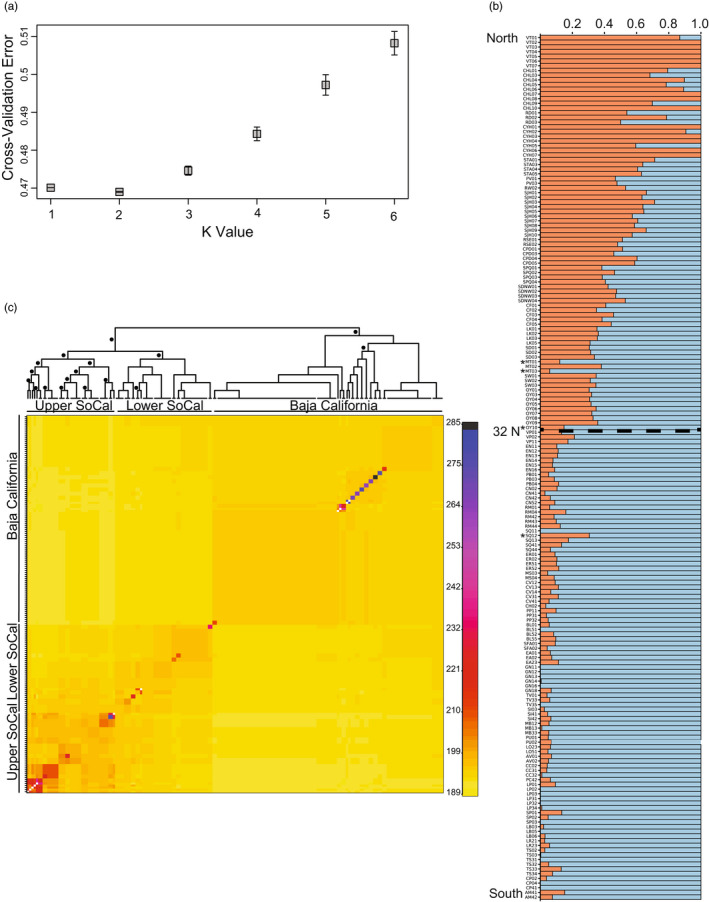
Genetic structure of the California gnatcatcher (a) Cross‐validation errors from Admixture analyses using all loci at values of K ranging from 1 to 6. The lowest error was found at K = 2. (b) Individual assignment plot from Admixture analysis of K = 2 with individuals arranged by collection location from north to south and a latitudinal cline in assignment proportions. (c) Coancestry heat map and cladogram from fineRADstructure. Dots on the cladogram denote nodes with greater than 95% bootstrap support, and the colors on the heatmap indicate pairwise coancestry between individuals, with blue and purple representing highest levels, red and orange intermediate levels, and yellow representing lowest levels of shared coancestry. The deepest split was between southern California and Baja birds

Admixture analysis marginally supported two clusters over a single cluster (K = 2 avg. cross‐validation = 0.469; K = 1 avg. cross‐validation 0.470; Figure 2a). The individual assignment plot at K = 2 showed a clinal pattern in assignment from north to south, with birds in San Diego County mostly 50% or more admixed with the southern cluster, and birds south of the international border more than 80% assigned to the southern cluster (Figure 2b). The resulting cladogram and coancestry matrix from fineRADstructure resolved two major groupings in the data, California and Baja, split at the international border (Figure 2c). Within the California group, individuals clustered geographically into two supported subclusters. “Upper SoCal” included birds from Ventura, Coyote Hills, Chino Hills, Santa Ana, San Joaquin Hills, and some individuals from Redlands. The “Lower SoCal” group included birds from Riverside and San Diego Counties, as well as from Rancho Palos Verdes and Redlands. The Baja clade lacked any geographically clustered substructure. Some evidence of recent migration was apparent. Two birds sampled in Mission Trails (MT1,3; San Diego County) and one from Otay River Park (OY10; San Diego County) clustered with the Baja group and one bird sampled in San Quintín (SQ1; Baja California) clustered with the Lower SoCal group.

In the rangewide dataset, neither Euclidean geographic distance nor human modified habitat cost distance were positively associated with genetic distance. The second result is perhaps not surprising, as human modified habitat mainly occurs in southern California (Figure 1). However, three of the climate variable distances (TMax, PrecipWQ and PrecipDQ) were positively associated with distance across the full range, along with a split between the California and Baja groups (Table S2). After stepwise elimination, the MRM model included the California and Baja groups and PrecipWQ (*R* ^2^ = 0.058, *p* ≤ 0.001). When analyzed separately, different patterns became apparent in the California and Baja groups. In the California group, Euclidean geographic distance and human modified habitat cost distance were both positively correlated with genetic distance, as were TMax, TMin, PrecipS and PrecipWQ (Table S2). The final MRM model included Modified Habitat Cost, TMax and PrecipWQ (*R* ^2^ = 0.23, *p* ≤ 0.001). In contrast, there was little detectable pattern in the Baja subgroup. PrecipS was the only variable associated with genetic distance in the Baja subgroup in the Mantel Test and MRM (*R* ^2^ = 0.012, *p* ≤ 0.042; Table S2).

### 3.3 Genetic climate associations

The full RDA model [genotypes ~ TMax + TMin + PrecipS + PrecipWQ +PrecipDQ] explained 1.2% of the total observed genetic variation (adjusted *R* ^2^ = 0.012, *p* < 0.001). All climate variables had inflation factors <10 and were not highly cross correlated (*r* ≤ 0.7) and were retained. There were 4 significant RDA axes (*p* ≤ 0.001).

**FIGURE 3:**
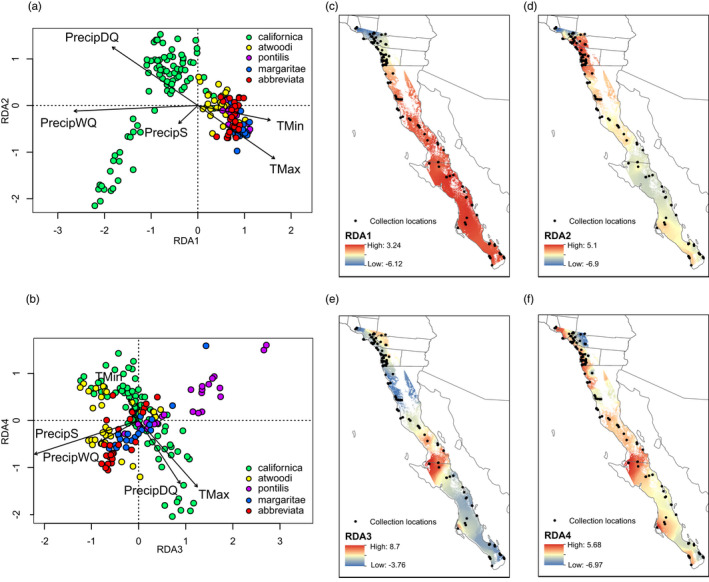
RDA results. Individual genotypes (colored by subspecies) and climatic variable loadings for (a) RDA axes 1 and 2, and (b) RDA axes 3 and 4. Point colors reflect hypothesis 1 subspecies designations. (c) Interpolated individual RDA 1 loadings, showing a distinction between southern California and Baja. (d) Individual RDA 2 loadings separated the northern‐most southern California samples in Ventura and Chino Hills and Coyote Hills [lower left of RDA plot (a)] most strongly from the rest of southern California. (e) RDA 3 distinguishes the mid‐peninsular *pontilis* group, and (f) RDA 4 loadings are mixed across much of the range, with an East–West cline apparent in samples north of 32°N

Mapping of RDA loading scores of individual multi‐locus genotypes revealed several spatial‐climatic patterns (Figure 3a,b). RDA 1 (35% of the variation) separated southern California from Baja (Figure 3a). Individual loadings followed a latitudinal cline centered near 32°N, with individuals to the south associated with higher minimum and maximum temperatures and individuals to the north associated with greater precipitation (Figure 3c, Table S3). RDA 2 (20% of the variation) was positively associated with PrecipDQ and negatively associated with TMax (Table S3). RDA 2 further separated the northern‐most samples (Ventura, Chino Hills and Coyote Hills) from the rest of the southern California samples (Figure 3a lower left quadrant; Figure 3d). RDA 3 (17% of the variation) was positively associated with TMax and negatively associated with seasonality in precipitation, with more seasonality at the northern range edge and in northern Baja, and less in the mid‐peninsular Vizcaíno Desert (Table S3, Figure 3e). RDA 4, negatively associated with both PrecipDQ and TMax, showed geographically widespread loadings across much of the range, with a strong east–west cline apparent in samples north of 32°N (Figure 3f).

**FIGURE 4:**
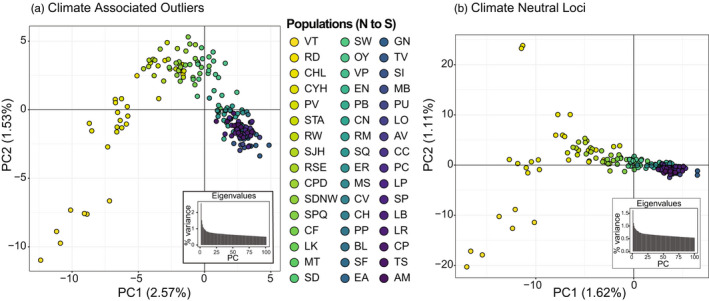
PCA plots of Axes 1 and 2 for (a) the climate associated outliers (6339), and (b) the climate neutral SNPS (77,786). Sampling locations are arranged from North (yellow) to South (dark purple)

The outlier analysis identified 6339 putatively adaptive (climate outlier) SNPs that loaded ±3 SD from the mean loading on one or more of the four significant RDA axes. Based on the strongest correlation between each outlier locus and climate variable, 1961 of the outlier SNPs were most strongly associated with PrecipWQ, 1466 with PrecipDQ, 1459 with TMax, 1046 with PrecipS, and 407 with TMin. Patterns across PC axes 1 and 2 in the climate outlier loci and the remaining climate neutral loci were relatively similar, with most Baja birds tightly clustered and southern California birds extending across PC 1 (Figure 4). However, individuals were more broadly spread across PC 2 in the climate outliers, and larger proportions of variation were explained by both PC 1 and PC 2 in the climate outlier loci (Figure 4a) than in the climate neutral set (Figure 4b). Finally, two clusters were best supported in the climate outlier loci based on BIC, while a single cluster had the lowest BIC in the climate neutral dataset (Figure S2), suggesting that climate outlier loci are contributing to population structure in the full genomic dataset.

### 3.4 Demographic history and contemporary diversity patterns

**FIGURE 5:**
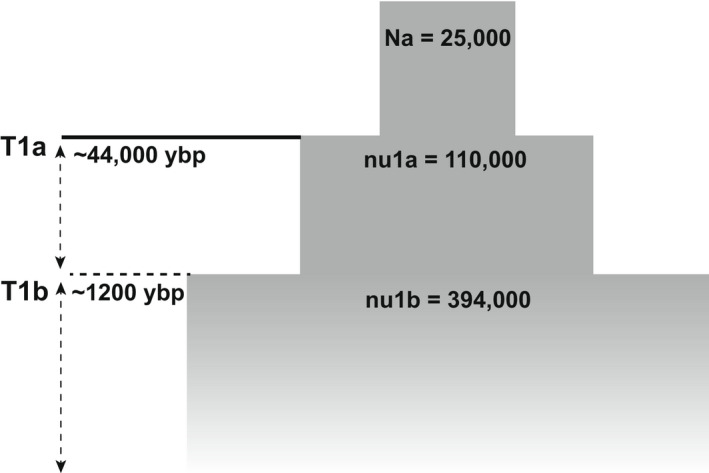
Demographic parameter estimates for the optimal three‐epoch model for *Polioptila californica*. Parameter estimates reported here are the ancestral population size (Na), ancient population size (nu1a), ancient population size (nu1b), the time in the past at which the first instantaneous size change happened (T1a), and the time in the past at which the second instantaneous size change happened (T1b); ybp, years before present

We found highest support for demographic models that involve a history of population expansion across the range of the gnatcatcher. The optimum model (highest likelihood score) was a three‐epoch model, which models multiple instantaneous size changes (Table S4) that occur as population expansion events. Using the calculated ϴ, we estimated the ancestral population (Na) to be ~25,000 with the first population expansion beginning at ~44,000 years before present (ybp) and again more recently at ~1200 ybp, consistent with late Pleistocene and Holocene expansion events (Figure 5). Models that included population bottlenecks had parameters that failed to converge, indicating poor fit, and the null model (no change) was consistently the model with the lowest likelihood score (Table S4). From the site frequency spectrum, the mean *Tajima's D* was estimated to be −1.59, consistent with a population expansion or a strong selective sweep (Table 2, Tajima, 1989). We also evaluated the demographic history separately for the California and Baja groups supported in the population structure analyses. However, within groups, most model comparisons failed to converge, suggesting that more data are likely needed to fully evaluate the demographic history within regional groups (results not shown).

Comparisons of most genetic diversity indices among sites, subspecies and groups were similar across the entire range, with the exception of sites in northern Baja (PB, ER, and SQ) and at the southern tip of Baja (SP, TS, and AM) where diversity indices were much higher (Figure S3). In addition, an increasing trend from northern to southern latitudes in the number of private alleles was observed across sites, subspecies, and genetic groups (Figure S4). Contemporary effective size (Ne) estimates were 695 (95% CIs: 451–2063) in southern California, and 531 (95% CIs: 281–5181) in Baja.

### 3.5 Subspecies distinctiveness

**FIGURE 6:**
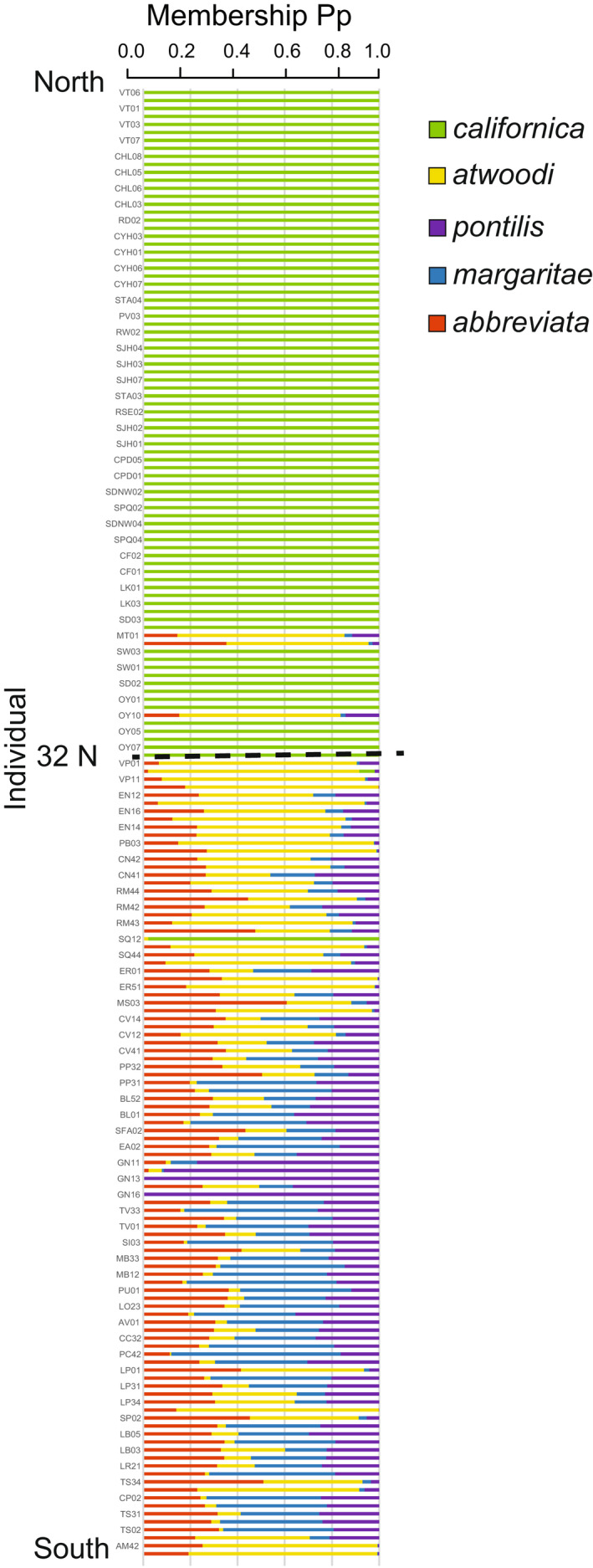
Discriminant Analysis of Principal Components (DAPC) posterior probabilities of assignments arranged from North to South with a dashed line at 32°N. Almost all individuals north of 32°N were assigned with high probability (>0.9) to *P. c. californica*. Almost no individuals south of 32°N could be assigned with high probability to their respective subspecies

In the DAPC analysis, the a‐score was optimized when including 12 principal components. Four discriminant functions were retained, accounting for 11.4% of the genetic variance. In the analysis of hypothesis 1 designations, *P. c. californica* was strongly discriminated on DA1 (Figure S5), with almost all individuals north of 32°N with greater than 90% posterior probability of assignment to the *P. c. californica* group (Table 3; Figure 6). As was found in the fineRADStructure co‐ancestry results, exceptions included 3 individuals collected in San Diego (1 in Otay River Valley, and 2 in Mission Trails Regional Park) that assigned most strongly to a mix of Baja California groups. One individual collected in San Quintín, Baja California had a high probability of assignment (98%) to the *P. c. californica* group. Among all the other birds sampled in Baja California, few had strong (>90%) assignment to any one of the southern groups, and the majority were substantially admixed (Table 3, Figure 6). Results using the 3 subspecies hypothesis of Atwood (1991; hypothesis 2) were very similar. Birds north of 32°N were almost all assigned to *P. c. californica*, and birds between 32° and 30°N had mixed assignments among all three subspecies (Figure S5). When all birds north of the 30°N boundary were grouped together, only 70% of birds assigned strongly to *P. c. californica* (Table 3). Similarly, pairwise Nei's genetic distances among all individuals were not significantly associated with either of the tested subspecies groupings (hypothesis 1 or 2; Table 1); although, as described in section 3.2, these did support a significant break between birds north of 32°N and all Baja California birds grouped.

**TABLE 3:** DAPC summary of probability of assignment to subspecies groups

Subspecies	Prop. of ind. with >90% assignment	Avg. assignment prob
Hypothesis 1 (Atwood, 1991; Mellink & Rea, 1994)		
*californica* (32°N)	0.96	0.96
*atwoodi*	0.03	0.58
*pontilis*	0.13	0.36
*margaritae*	0.00	0.44
*abbreviata*	0.00	0.3
Hypothesis 2 (Atwood, 1991)		
*californica* (30°N)	0.70^a^	0.81
*margaritae*	0.05	0.67
*abbreviata*	0.00	0.33

^a^ All individuals with >90% assignment were sampled North of 32°N.

Hierarchical AMOVA tests suggested that a small but statistically significant amount of genetic variation was partitioned among subspecies for both hypothesis 1 and hypothesis 2 (Table S5). Generally, higher Φ_CT_ (among subspecies) and Φ_SC_ (among populations within subspecies) estimates were obtained with the set of climate‐associated loci than with all loci. A slightly larger proportion of variation was partitioned among subspecies using hypothesis 1 groupings (All loci 0.44%; Climate loci 0.98%) than hypothesis 2 groupings (All loci 0.40%; Climate loci 0.86%; Table S5). However, in all tests, the proportion of variation partitioned among populations within subspecies was higher than that among subspecies (Table S5). All hypothesis 1 pairwise comparisons between *P. c. californica* and all other subspecies were significantly different in both sets of loci (all loci or climate loci). Only one other subspecies, *P. c. atwoodi*, was significantly different from geographically neighboring subspecies, and only in the climate‐associated loci. For hypothesis 2, pairwise tests were significant between *P. c. californica* and both *P. c. margaritae* and *P. c. abbreviata* in the climate loci, but only *P. c. margaritae* in the full set of loci. (Table S5).

## 4 DISCUSSION

Our data support two genetic groups within the California gnatcatcher, a southern California group and a Baja group with admixture, suggesting either a recent evolutionary origin or a stable tension between zones of adaptive differentiation countered by occasional gene flow (Barton & Hewitt, 1985). The difference between the California and Baja groups was statistically supported by the fineRADstructure analysis, genetic distance analyses, the DAPC, and in consistently significant partitioning of variation between *P. c. californica* and all other subspecies in the AMOVA tests. A small number of recent migrants, and higher genetic diversity in northern Baja are consistent with recent dispersal and admixture between these groups in San Diego County and northern Baja California. Patterns of differentiation were stronger in climate outlier loci than the remainder of climate neutral loci which could be indicative of adaptive differences in the two groups.

### 4.1 Genetic differentiation and climate adaptation

Patterns of genetic differentiation were different in the two regional genetic groups. In Baja, high admixture across large geographic distances was apparent, and only seasonality in precipitation was associated with genetic differentiation. In southern California, differentiation was strongly associated with both habitat fragmentation and with clines in climate, particularly in precipitation and minimum winter temperature. Previously, high genetic connectivity and evidence of occasional long‐distance dispersal throughout much of southern California were found with microsatellites (Vandergast et al., 2019). The exception was in the northern‐most portion of the range (Ventura, Redlands, Chino Hills, Palos Verdes, Coyote Hills, San Joaquin Hills), where habitat patches are more fragmented (Vandergast et al., 2019). In these locations, where less than 10 percent of the surrounding habitat was suitable for gnatcatchers, genetic divergence was highest, and diversity was lowest (Vandergast et al., 2019). The California gnatcatcher was originally listed as threatened in southern California due to declines in populations linked to extensive loss and fragmentation of coastal sage scrub habitat (Atwood, 1993), with estimates of habitat loss between the 1940s and 1980s of 70%–90% (Westman, 1981). Habitat reduction was likely most severe in Los Angeles, San Bernardino, Ventura and Riverside Counties (Atwood & Bontrager, 2020). Similarly, habitat loss in northern Baja California may be contributing to, or reinforcing, the genetic differentiation apparent at the international border. As early as the 1990s loss of habitat due to agriculture, grazing, housing and urban development rendered connection between southern California and Baja populations tenuous (Atwood & Bontrager, 2020; USFWS, 1993). Anecdotally, aggregations of gnatcatchers are sparse in this region. Our survey efforts in this area suggest that habitat and aggregations of birds were more thinly distributed in northern Baja than further south. These observations echo those of Mellink and Rea (1994) who noted that aggregations of gnatcatchers were sporadic in the region between the international border and El Rosario, Baja California, and that many areas of suitable‐looking habitat did not support birds.

Gnatcatchers in southern California are associated with generally lower temperatures and higher precipitation than in Baja. In addition, some of the northern‐most populations contain unique genetic variation associated with drier and/or warmer summers and more seasonal precipitation. These differences may be of adaptive significance and warrant further investigation. Range limitations in California gnatcatchers are associated with some combination of low winter temperatures and winter rainfall, suggesting physiological limitations in cold tolerance in this species (Mock, 1998). Differences in body mass and plumage coloration occur across the range, both of which can indicate adaptive responses to precipitation and temperature. Southern California males and females were 10% and 8% larger than males and females from the Cape region of Baja, respectively (Atwood & Bontrager, 2020). Similarly, Atwood (1991) Figure 4 showed that a measure of size (midtoe plus tarsal length) was generally larger in museum skins from the northern‐most “Los Angeles” group than elsewhere in the range. Larger body mass in birds is related to cold tolerance both in terms of basal metabolic rate and summit metabolic rate, or the maximum cold‐induced metabolic rate, which is important for residency in colder climates (Swanson & Garland, 2009). Both body size and metabolic rates are in turn informed by phylogenetic relationships among birds and hypothesized to play a role in adaptive radiation (Swanson & Garland, 2009). High winter mortality rates in California gnatcatchers may exert strong selective pressure for increased cold tolerance in outlying populations. Phenotypic change in birds in response to climate can be rapid, and studies have documented widespread reductions in body size attributed to a warming climate (Van Buskirk et al., 2010; Weeks et al., 2020). In contrast, an assemblage of birds in central California chaparral systems (similar to the gnatcatcher's northern range) are increasing in body size (Goodman et al., 2012). This unexpected pattern may be a selective response to greater climate variability (more extreme precipitation events) or to climate‐related patterns of primary productivity. Southern California birds also have darker back plumage, which might relate to higher precipitation and cooler temperatures (e.g., revised Gloger's Rule, see Marcondes et al., 2021). Although an annotated genome for the California gnatcatcher is not currently available, additional efforts could map outlier loci to annotated genomes of other songbirds, and explore the functions of putatively climate‐associated genes. Such efforts could be helpful in determining if these patterns are due to local selection, genetic drift, or influenced by both. Regardless, from a conservation perspective, these patterns emphasize the importance of protecting these outlying populations.

### 4.2 Historical range expansion

Previously published genetic and species distribution analyses were suggestive of a northward range expansion in gnatcatchers (Zink et al., 2000, 2013), likely following the last glacial maximum (Zink et al., 2013). We found that private alleles were highest in the southern peninsula and generally decreased moving northward, consistent with the southern region being an older and more diverse ancestral population. However, we found support for multiple expansion events, roughly timed at the end of the Pleistocene and Holocene. Additionally, we found two regions of comparatively high heterozygosity and nucleotide diversity: the southern tip of Baja, and northern Baja. This pattern could be consistent with an initial northward range expansion from the south, followed by a period of contraction to multiple refugia and subsequent recontact in northern Baja. Previously published climate envelope models at the last glacial maximum appear to show small regions of suitable climate space for gnatcatchers in coastal Los Angeles and San Diego, as well as a large area of southern Baja (Zink et al., 2013; Figure 9).

Contemporary effective population sizes, based on linkage disequilibrium, are much lower than historical estimates derived from the JSFS, with 95% confidence intervals ranging from the low hundreds to low thousands. The large difference in size estimates obtained with these two methods could reflect the evolutionary time scale of the model estimates versus the very recent time scale of linkage disequilibrium, which dissipates over a few generations (Waples, 2005). Previously reported (linkage‐based) estimates of effective size in the California portion of the range estimated with microsatellite markers (Vandergast et al., 2019) are similar to those presented here, with overlapping confidence intervals. Contemporary effective size estimates are above the recommended sizes of 50–100 to avoid inbreeding depression, and could be above or below the recommended 500–1000 to preserve evolutionary and adaptive potential (Frankham et al., 2014; Franklin, 1980). Development throughout southern California is more extensive than throughout most of Baja (with the exception of the international border and Cape regions). The link between development and genetic distances in southern California suggests that efforts to retain and improve connectivity could be useful in maintaining high effective population size, while continued monitoring of aggregations throughout the range would allow for early detection of declines in abundance.

### 4.3 Subspecies designations

Our four analyses found genomic differences between *P. c. californica* and all other birds sampled in Baja but did not strongly support other hypothesized subspecies breaks in Baja. Applying a definition of a subspecies as a breeding population or collection of populations that occupies a distinct segment of the geographic range of the species and that is measurably distinct in phenotype, genotype or a combination of these traits (e.g., Haig & D'Elia, 2010), we find genomic support for retaining *P. c. californica* as a distinct subspecies. We found stronger support for a genetic boundary or contact zone between *californica* and its neighboring subspecies near 32°N as hypothesized by Mellink and Rea (1994), rather than 30°N suggested by Atwood (1991). All four tests supported a distinct *P. c. californica* at 32°N, while only the two pairwise AMOVA tests were significant at 30°N. The lack of specificity in the AMOVA may be due to the smaller number of individuals sampled in the region between 30°–32°N compared to north of the border, while the two more spatially explicit approaches resolve this boundary with greater precision. If the few mis‐assigned birds in San Diego County and northern Baja are recent migrants, this could suggest a relatively broad contact zone with at least some recent movement and gene flow.

Conversely, our data indicate high genetic similarity and connectivity across the remainder of the Baja Peninsula, despite the large geographic area and range of climatic conditions encompassed therein. However, as previously noted, genetic patterns are not always congruent with morphological traits. In cases where morphological adaptation has occurred recently or in the presence of gene flow, the majority of the genome is unlikely to show high differentiation (Fitzpatrick, 2010). This is likely the case for the California gnatcatcher, where recent range expansion is supported, and ongoing gene flow also seems likely. Similarly, Patten (2015) pointed out that when a subspecies is originally defined by its morphological diagnosability (sensu Patten & Unitt, 2002), phenotype as well as genotype should be accounted for. Given our genetic results, a reanalysis of the morphological traits originally used to differentiate among these Baja subspecies may be warranted.

### 4.4 Managing the California gnatcatcher

Based on the shallow divergences concordant with areas of highest habitat loss, patterns of evolutionarily recent range expansions, and indications here and in previous studies that gnatcatchers are able to disperse long distances (Bailey & Mock, 1998; Vandergast et al., 2019), the California gnatcatcher may benefit from habitat restoration to enhance connectivity where habitat is becoming limited. Given that the northern‐most sampled populations of Ventura, Chino Hills, and Coyote Hills appear to harbor unique genetic variation associated with seasonal extremes in precipitation and temperatures, maintaining and restoring habitat in the most northern and eastern range edges could help preserve this reservoir to facilitate continued range expansion, particularly as warmer temperatures and more frequent and intense drought with variable and extreme precipitation events are predicted throughout southern California (Berg & Hall, 2015; Cayan et al., 2010; Kam & Sheffield, 2016; Swain, 2015). At least in some areas of southern California, climate change and development have already led to a loss of gnatcatcher habitat (Hulton VanTassel et al., 2017). Efforts to identify and protect future suitable habitat, and protect existing adaptive potential that allows for range expansion, may be important strategies to ensure long‐term persistence of the California gnatcatcher.

## CONFLICT OF INTEREST

The authors declare that there is no conflict of interest.

## Supporting information

Tables S1‐S5 and Figures S1‐S5

## Data Availability

Raw data are accessible as an NCBI Sequence Read Archive (https://www.ncbi.nlm.nih.gov/bioproject/PRJNA844114). Genotype calls and location data are available as a USGS ScienceBase Data Release (https://doi.org/10.5066/P9MB2YE2; (Wood & Vandergast, 2022)).
