# Supplementary material for: Subspecies differentiation and range‐wide genetic structure are driven by climate in the California gnatcatcher, a flagship species for coastal sage scrub conservation
Source: Evol Appl. 2022 Jun 29;15(7):1201–17. doi: 10.1111/eva.13429 (PMC9309440; doi:10.1111/eva.13429)
Supplement: Supplementary file 1 — Tables S1‐S5 and Figures S1‐S5 [file EVA-15-1201-s001.pdf]

Table S1: Samples and sequencing coverage and quality.

Sample	Latitude	Longitude	Sub_mellink	Sub_atwood	Country	BAM_reads	BAM_reads_kept	BAM_kept_frac	Coverage	Loci_Present_R80	Loci_Absent_R80	Perc_Missing_Data
ER01	30.11486	-115.65182	atwoodi	californica	MX	2315199	2132304	0.921	19	83853	256	0.3
CN52	31.11516	-116.19340	atwoodi	californica	MX	2654823	2435100	0.917	22	83887	222	0.3
GN12	27.92040	-113.93051	pontilis	margaritae	MX	754277	694038	0.92	9	83777	332	0.4
CH02	29.34689	-114.32795	pontilis	margaritae	MX	841372	774944	0.921	9	83797	312	0.4
AM41	23.04663	-110.10189	abbreviata	abbreviata	MX	1266960	1169928	0.923	12	83791	318	0.4
LB06	23.68921	-109.70243	abbreviata	abbreviata	MX	1726614	1576544	0.913	17	83739	370	0.4
SJH01	33.56909	-117.81666	californica	californica	USA	3969025	3685403	0.929	16	83785	324	0.4
EA01	28.01590	-113.41917	pontilis	margaritae	MX	3009921	2753807	0.915	22	83799	310	0.4
BL01	28.89836	-113.53063	pontilis	margaritae	MX	4509313	4102934	0.91	29	83731	378	0.4
CHL01	34.03768	-117.86789	californica	californica	USA	4805654	4442521	0.924	30	83809	300	0.4
GN13	27.91835	-113.93023	pontilis	margaritae	MX	6887573	6358612	0.923	38	83763	346	0.4
BL51	28.96928	-113.55279	pontilis	margaritae	MX	536872	480119	0.894	8	83703	406	0.5
GN11	27.92041	-113.93052	pontilis	margaritae	MX	1297751	1204090	0.928	11	83706	403	0.5
EN14	31.73150	-116.72320	atwoodi	californica	MX	1229721	1130820	0.92	12	83695	414	0.5
LP01	24.13086	-110.27844	abbreviata	abbreviata	MX	1479987	1348411	0.911	15	83669	440	0.5
CF03	32.97527	-117.12403	californica	californica	USA	3299188	3051026	0.925	15	83659	450	0.5
SPQ03	33.02724	-116.79164	californica	californica	USA	3195915	2938743	0.92	19	83728	381	0.5
CF02	32.94796	-117.11882	californica	californica	USA	6877012	6320436	0.919	25	83703	406	0.5
GN16	27.91515	-113.92637	pontilis	margaritae	MX	5968172	5527100	0.926	29	83647	462	0.5
CHL06	34.08507	-117.79923	californica	californica	USA	5501774	5029461	0.914	34	83654	455	0.5
SDNW03	33.09418	-117.27124	californica	californica	USA	11059628	10158675	0.919	48	83680	429	0.5
CV12	29.75688	-114.74582	pontilis	margaritae	MX	796525	738511	0.927	8	83644	465	0.6
EN15	31.73426	-116.70351	atwoodi	californica	MX	599119	524027	0.875	10	83573	536	0.6
EN11	31.74886	-116.55513	atwoodi	californica	MX	1270836	1162390	0.915	11	83618	491	0.6
GN14	27.91831	-113.93025	pontilis	margaritae	MX	1277833	1183279	0.926	12	83607	502	0.6
CC32	25.09746	-111.70074	margaritae	margaritae	MX	1233365	1128302	0.915	13	83610	499	0.6
CP02	23.46482	-109.45225	abbreviata	abbreviata	MX	1284834	1192120	0.928	12	83627	482	0.6
BL52	28.96943	-113.55577	pontilis	margaritae	MX	1634113	1510670	0.924	13	83568	541	0.6
EN13	31.74475	-116.55481	atwoodi	californica	MX	1288562	1187526	0.922	14	83588	521	0.6
CYH05	33.89697	-117.90155	californica	californica	USA	4101207	3754652	0.915	22	83630	479	0.6
CF05	32.91394	-117.17600	californica	californica	USA	4090862	3775828	0.923	26	83582	527	0.6
LK03	32.82292	-116.91124	californica	californica	USA	11529487	10675098	0.926	53	83593	516	0.6
CV41	29.66061	-114.64888	pontilis	margaritae	MX	1014954	943592	0.93	11	83489	620	0.7
EN16	31.73548	-116.70704	atwoodi	californica	MX	1727155	1575144	0.912	16	83501	608	0.7
AV01	25.51563	-111.08194	margaritae	margaritae	MX	3345870	3100439	0.927	18	83519	590	0.7
EA02	28.00994	-113.43535	pontilis	margaritae	MX	5454006	5014429	0.919	27	83499	610	0.7
CN02	31.06340	-116.15399	atwoodi	californica	MX	631350	577151	0.914	8	83430	679	0.8
LP34	24.09626	-110.00508	abbreviata	abbreviata	MX	543971	503408	0.925	8	83463	646	0.8
AM42	23.05086	-110.09043	abbreviata	abbreviata	MX	1588114	1458619	0.918	15	83452	657	0.8
CYH06	33.89749	-117.89587	californica	californica	USA	6267119	5840679	0.932	24	83409	700	0.8
CHL04	34.08213	-117.79769	californica	californica	USA	6720396	6255137	0.931	34	83451	658	0.8
SI03	27.30205	-112.88842	margaritae	margaritae	MX	1580146	1440137	0.911	13	83377	732	0.9
CHL03	34.05846	-117.83445	californica	californica	USA	4394360	3972110	0.904	25	83370	739	0.9
STA01	33.76797	-117.76434	californica	californica	USA	6300132	5860964	0.93	29	83377	732	0.9
LR21	23.61761	-109.62351	abbreviata	abbreviata	MX	760032	698079	0.918	9	83295	814	1
RM43	30.95202	-115.99375	atwoodi	californica	MX	1566375	1436218	0.917	12	83291	818	1
SJH06	33.61632	-117.81477	californica	californica	USA	1629036	1501444	0.922	12	83276	833	1

CYH04	33.89820	-117.90009	californica	californica	USA	1792641	1639926	0.915	14	83275	834	1
ER52	30.05121	-115.78249	atwoodi	californica	MX	1575069	1453310	0.923	14	83272	837	1
SPQ04	33.02724	-116.79164	californica	californica	USA	4061385	3743399	0.922	25	83255	854	1
EA23	27.78724	-113.37021	pontilis	margaritae	MX	5108453	4716249	0.923	22	83278	831	1
CF01	32.94700	-117.11869	californica	californica	USA	6177605	5720325	0.926	23	83229	880	1
GN18	27.92191	-113.92242	pontilis	margaritae	MX	5301080	4810439	0.907	33	83259	850	1
CHL10	34.00318	-118.04662	californica	californica	USA	9898513	9144293	0.924	48	83273	836	1
CC02	25.00568	-111.65279	margaritae	margaritae	MX	2011626	1868042	0.929	15	83225	884	1.1
SJH03	33.61978	-117.90627	californica	californica	USA	2876221	2664473	0.926	14	83222	887	1.1
SD02	32.73460	-117.14315	californica	californica	USA	4413940	4085095	0.925	18	83209	900	1.1
LK02	32.84948	-116.91024	californica	californica	USA	8043245	7421365	0.923	38	83181	928	1.1
BL55	28.97284	-113.55405	pontilis	margaritae	MX	1838495	1707284	0.929	16	83070	1039	1.2
AV02	25.51705	-111.08139	margaritae	margaritae	MX	2637680	2446094	0.927	18	83097	1012	1.2
CHL09	33.87522	-117.67494	californica	californica	USA	4007094	3699262	0.923	30	83122	987	1.2
CYH03	33.90122	-117.94667	californica	californica	USA	4783748	4361755	0.912	26	83078	1031	1.2
LK05	32.82255	-116.90704	californica	californica	USA	9614318	8742404	0.909	37	83104	1005	1.2
CHL08	34.09212	-117.81593	californica	californica	USA	5654804	5201854	0.92	30	83052	1057	1.3
SFA02	28.63070	-113.17973	pontilis	margaritae	MX	1670415	1534724	0.919	18	82938	1171	1.4
EN12	31.74705	-116.55031	atwoodi	californica	MX	912992	842923	0.923	10	82861	1248	1.5
CP04	23.43506	-109.43238	abbreviata	abbreviata	MX	1322759	1219624	0.922	13	82862	1247	1.5
LP02	24.13083	-110.27844	abbreviata	abbreviata	MX	1570318	1447474	0.922	16	82871	1238	1.5
CN42	31.11768	-116.19622	atwoodi	californica	MX	561253	518402	0.924	8	82785	1324	1.6
VT05	34.23312	-118.88361	californica	californica	USA	6082691	5564622	0.915	29	82790	1319	1.6
SPQ01	33.08780	-116.98516	californica	californica	USA	5955439	5527168	0.928	29	82644	1465	1.7
LP31	24.10197	-110.30876	abbreviata	abbreviata	MX	975588	893144	0.915	10	82600	1509	1.8
RSE02	33.59481	-117.06093	californica	californica	USA	4324615	4007983	0.927	24	82604	1505	1.8
LP03	24.13131	-110.28264	abbreviata	abbreviata	MX	701595	651979	0.929	8	82440	1669	2
CN41	31.10773	-116.18881	atwoodi	californica	MX	828953	760610	0.918	11	82420	1689	2
SQ11	30.48703	-116.01364	atwoodi	californica	MX	825847	747298	0.905	10	82442	1667	2
CHL05	34.08747	-117.79953	californica	californica	USA	6116094	5580980	0.913	32	82452	1657	2
ER51	30.05166	-115.78116	atwoodi	californica	MX	1684654	1554201	0.923	15	82348	1761	2.1
CF04	32.94772	-117.08994	californica	californica	USA	3612654	3333517	0.923	21	82330	1779	2.1
ER02	30.11230	-115.65813	atwoodi	californica	MX	4447694	4065032	0.914	20	82323	1786	2.1
CPD04	33.40305	-117.59066	californica	californica	USA	4692592	4346436	0.926	22	82214	1895	2.3
CPD01	33.28674	-117.23162	californica	californica	USA	5861512	5397711	0.921	20	82160	1949	2.3
SFA01	28.63611	-113.17781	pontilis	margaritae	MX	6336512	5838980	0.921	29	82156	1953	2.3
LO23	25.99105	-111.36074	margaritae	margaritae	MX	7174603	6684184	0.932	27	82173	1936	2.3
TV35	27.42230	-112.52792	margaritae	margaritae	MX	1304501	1193599	0.915	14	82084	2025	2.4
SPQ02	33.09177	-116.98210	californica	californica	USA	4097368	3791356	0.925	26	82122	1987	2.4
LP32	24.09931	-110.30333	abbreviata	abbreviata	MX	614382	558691	0.909	8.05	82019	2090	2.5
CPD03	33.31463	-117.48338	californica	californica	USA	5101841	4714685	0.924	22	81850	2259	2.7
PV01	33.73862	-118.37379	californica	californica	USA	6786330	6308246	0.93	39	81783	2326	2.8
CV31	29.73012	-114.69794	pontilis	margaritae	MX	3780516	3487040	0.922	21	81616	2493	3
SDNW02	33.09442	-117.22833	californica	californica	USA	5307413	4909104	0.925	31	81603	2506	3
SQ12	30.47877	-116.01384	atwoodi	californica	MX	2040443	1882947	0.923	14	81516	2593	3.1
PU02	26.37969	-111.70442	margaritae	margaritae	MX	2714460	2522205	0.929	18	81539	2570	3.1
PP11	29.04934	-114.13503	pontilis	margaritae	MX	2934251	2704319	0.922	21	81430	2679	3.2
CPD05	33.38705	-117.55415	californica	californica	USA	2932230	2735634	0.933	16	81314	2795	3.3
SP03	24.03724	-109.98192	abbreviata	abbreviata	MX	1731915	1599799	0.924	18	81177	2932	3.5
CYH02	33.89958	-117.94592	californica	californica	USA	3303356	3058697	0.926	15	80887	3222	3.8

RM04	30.95386	-116.07801	atwoodi	californica	MX	4012038	3712321	0.925	22	80896	3213	3.8
TS02	23.40215	-110.20098	abbreviata	abbreviata	MX	2274255	2105366	0.926	19	80542	3567	4.2
PB03	31.70017	-116.67387	atwoodi	californica	MX	735950	674662	0.917	9	80327	3782	4.5
CV13	29.75881	-114.74669	pontilis	margaritae	MX	2598807	2416164	0.93	18	80333	3776	4.5
SP02	24.03724	-109.98193	abbreviata	abbreviata	MX	1387868	1277665	0.921	15	80164	3945	4.7
SI41	27.30226	-112.89854	margaritae	margaritae	MX	2363082	2177259	0.921	17	80100	4009	4.8
RW02	33.71312	-117.36759	californica	californica	USA	4690826	4320320	0.921	20	80066	4043	4.8
RD01	34.08917	-117.12703	californica	californica	USA	11350576	10451665	0.921	60	79936	4173	5
SJH05	33.63315	-117.77039	californica	californica	USA	7341072	6809089	0.928	34	79800	4309	5.1
MB13	26.64249	-111.84485	margaritae	margaritae	MX	2173312	1966233	0.905	18	79655	4454	5.3
VT02	34.23595	-118.87018	californica	californica	USA	4396234	4081453	0.928	29	79518	4591	5.5
OY08	32.55926	-117.10303	californica	californica	USA	6815806	6328877	0.929	39	79509	4600	5.5
SQ13	30.45981	-116.03058	atwoodi	californica	MX	998978	920139	0.921	10	79228	4881	5.8
CHL07	34.09365	-117.81303	californica	californica	USA	7103208	6489582	0.914	36	79222	4887	5.8
SJH08	33.61886	-117.81639	californica	californica	USA	1745221	1621581	0.929	14	79154	4955	5.9
MB33	26.89119	-111.95200	margaritae	margaritae	MX	2990702	2742153	0.917	18	79076	5033	6
VT06	34.26959	-118.85864	californica	californica	USA	8488027	7841071	0.924	46	79035	5074	6
VT03	34.23299	-118.87979	californica	californica	USA	6932221	6246955	0.901	33	78894	5215	6.2
CV14	29.76039	-114.74666	pontilis	margaritae	MX	1381396	1284804	0.93	13	78792	5317	6.3
SJH10	33.56947	-117.83468	californica	californica	USA	1785907	1639661	0.918	14	78616	5493	6.5
PB04	31.69784	-116.67244	atwoodi	californica	MX	1467284	1339880	0.913	13	78644	5465	6.5
CYH01	33.89957	-117.94303	californica	californica	USA	4835470	4456907	0.922	18	78591	5518	6.6
RM44	30.97790	-116.12269	atwoodi	californica	MX	1184030	1099797	0.929	12	78130	5979	7.1
RD02	34.03747	-117.36881	californica	californica	USA	13416290	12384255	0.923	52	77999	6110	7.3
PV03	33.74692	-118.41233	californica	californica	USA	3079757	2843142	0.923	20	77790	6319	7.5
SW01	32.73581	-116.92096	californica	californica	USA	6019231	5536562	0.92	33	77537	6572	7.8
OY04	32.54121	-117.09152	californica	californica	USA	8432545	7799397	0.925	43	77578	6531	7.8
TS34	23.48900	-110.27150	abbreviata	abbreviata	MX	2107583	1939602	0.92	18	77430	6679	7.9
MS03	30.04653	-115.29558	atwoodi	californica	MX	1187542	1099245	0.926	10	77145	6964	8.3
VP11	32.12933	-116.88277	atwoodi	californica	MX	1842720	1704178	0.925	17	77156	6953	8.3
TS31	23.44394	-110.24040	abbreviata	abbreviata	MX	994121	921995	0.927	12	76968	7141	8.5
STA04	33.79824	-117.79295	californica	californica	USA	3266112	2997904	0.918	17	76974	7135	8.5
SDNW04	33.06265	-117.09718	californica	californica	USA	6810162	6262096	0.92	24	76911	7198	8.6
LK01	32.85339	-116.90735	californica	californica	USA	4042931	3740179	0.925	23	76301	7808	9.3
SJH07	33.61815	-117.81233	californica	californica	USA	1566760	1453068	0.927	13	76025	8084	9.6
VT04	34.23299	-118.87979	californica	californica	USA	4258503	3937230	0.925	30	75933	8176	9.7
MT03	32.78585	-117.04916	californica	californica	USA	1023729	945286	0.923	10	75829	8280	9.8
VT07	34.19620	-118.93392	californica	californica	USA	7080752	6534026	0.923	32	75906	8203	9.8
RM42	30.95623	-115.88009	atwoodi	californica	MX	805541	736847	0.915	8	75807	8302	9.9
MT01	32.78788	-117.08171	californica	californica	USA	1233827	1145868	0.929	12	75756	8353	9.9
MB12	26.64248	-111.84481	margaritae	margaritae	MX	1109270	1006258	0.907	11	75508	8601	10.2
SP01	24.03851	-109.98055	abbreviata	abbreviata	MX	2559341	2351935	0.919	20	75555	8554	10.2
SQ44	30.42034	-115.92998	atwoodi	californica	MX	789293	725265	0.919	10	75211	8898	10.6
TV33	27.42633	-112.53265	margaritae	margaritae	MX	5420383	4927874	0.909	33	75135	8974	10.7
RSE01	33.58880	-117.02632	californica	californica	USA	2396869	2223130	0.928	13	75050	9059	10.8
STA05	33.69782	-117.69029	californica	californica	USA	7049688	6529179	0.926	24	74354	9755	11.6
RD03	34.08655	-117.13602	californica	californica	USA	5587545	5120421	0.916	23	74237	9872	11.7
TS03	23.39769	-110.19743	abbreviata	abbreviata	MX	776246	659260	0.849	12	74195	9914	11.8
PP31	29.04702	-114.13339	pontilis	margaritae	MX	1373390	1262470	0.919	13	74048	10061	12
MS04	29.98589	-115.20939	atwoodi	californica	MX	1530027	1382618	0.904	15	73767	10342	12.3

PB01	31.70626	-116.68479	atwoodi	californica	MX	814243	752366	0.924	11	73613	10496	12.5
VT01	34.23481	-118.87216	californica	californica	USA	5501787	5100889	0.927	29	73339	10770	12.8
LR23	23.63580	-109.66587	abbreviata	abbreviata	MX	1016267	930965	0.916	11	73230	10879	12.9
STA03	33.61182	-117.54934	californica	californica	USA	4901094	4514935	0.921	20	72855	11254	13.4
LO51	25.99923	-111.38458	margaritae	margaritae	MX	4736874	4411306	0.931	32	72306	11803	14
OY10	32.58221	-116.90742	californica	californica	USA	5277899	4856715	0.92	32	72339	11770	14
TS32	23.45606	-110.24864	abbreviata	abbreviata	MX	895339	830803	0.928	10	72252	11857	14.1
CC31	25.34053	-111.62283	margaritae	margaritae	MX	1992373	1836983	0.922	17	71688	12421	14.8
VP02	32.19736	-116.45505	atwoodi	californica	MX	1127166	1028942	0.913	12	71495	12614	15
CP41	23.49605	-109.47344	abbreviata	abbreviata	MX	3230985	2970792	0.919	23	71469	12640	15
LB03	23.68585	-109.70408	abbreviata	abbreviata	MX	699232	642919	0.919	8	70912	13197	15.7
TS33	23.48775	-110.27113	abbreviata	abbreviata	MX	957907	868039	0.906	10	70809	13300	15.8
SJH04	33.63646	-117.76901	californica	californica	USA	2585961	2382543	0.921	21	70679	13430	16
PC42	24.80337	-112.10800	margaritae	margaritae	MX	4004672	3720905	0.929	26	70654	13455	16
TV01	27.36694	-112.72062	margaritae	margaritae	MX	1512333	1403394	0.928	14	70216	13893	16.5
SJH09	33.60701	-117.75326	californica	californica	USA	6185248	5703730	0.922	27	70122	13987	16.6
OY09	32.58192	-116.91070	californica	californica	USA	9888184	9157575	0.926	37	70100	14009	16.7
OY03	32.59864	-116.95568	californica	californica	USA	6072106	5663937	0.933	35	69948	14161	16.8
PU01	26.34996	-111.74466	margaritae	margaritae	MX	5146300	4664577	0.906	34	69389	14720	17.5
OY06	32.68685	-116.85622	californica	californica	USA	3283251	3020196	0.92	16	69243	14866	17.7
OY01	32.61498	-116.93831	californica	californica	USA	7383489	6851334	0.928	44	69262	14847	17.7
SDNW01	33.13546	-117.30137	californica	californica	USA	3365033	3092088	0.919	22	69101	15008	17.8
CYH07	33.89430	-117.90589	californica	californica	USA	6449355	5943378	0.922	24	69147	14962	17.8
RM01	30.96077	-115.80289	atwoodi	californica	MX	1144059	1036123	0.906	13	68521	15588	18.5
SD03	32.79335	-117.18508	californica	californica	USA	5255393	4883448	0.929	30	68583	15526	18.5
SW02	32.73867	-116.92521	californica	californica	USA	7932067	7337489	0.925	47	68529	15580	18.5
SD01	32.73460	-117.14315	californica	californica	USA	7259582	6648496	0.916	34	68127	15982	19
SQ41	30.41872	-115.92711	atwoodi	californica	MX	664573	589634	0.887	10	67162	16947	20.1
OY05	32.57329	-117.03302	californica	californica	USA	6586086	6098345	0.926	22	66890	17219	20.5
LB05	23.68922	-109.70246	abbreviata	abbreviata	MX	680191	617469	0.908	9	66786	17323	20.6
SJH02	33.57012	-117.81521	californica	californica	USA	4336950	4010435	0.925	16	66405	17704	21
OY07	32.55895	-117.10386	californica	californica	USA	4180528	3815609	0.913	25	64613	19496	23.2
SW03	32.75339	-116.89552	californica	californica	USA	5706097	5276374	0.925	40	64610	19499	23.2
VP01	32.19926	-116.46505	atwoodi	californica	MX	778654	715335	0.919	8.41	63299	20810	24.7
PP32	29.04966	-114.13300	pontilis	margaritae	MX	1801176	1667485	0.926	15	61943	22166	26.4
SI42	27.30101	-112.90992	margaritae	margaritae	MX	1983096	1833452	0.925	12	60529	23580	28
MT02	32.79275	-117.07409	californica	californica	USA	4566670	4231303	0.927	21	58990	25119	29.9

Table S2. Results of Mantel tests and Multiple Regression of Distance Matrices for the full range, and within the southern California (CA) and Baja California (Baja) Groups. Pairwise Nei's genetic distance (Nei's GD) was estimated between all individuals. Habitat and climate distances included Euclidean geographic distance (Euc. Dist.), human modified habitat cost distance (Mod. Habitat Cost), maximum temperature of the warmest month (TMax), minimum temperature of the coldest month (Tmin), precipitation of the wettest quarter (PrecipWQ), precipitation of the driest quarter (PrecipDQ) and precipitation seasonality (PrecipS).

**Mantel Test**				**Multiple Regression of Matrices**			
**Full Range**		**Mantel R**	**p-value**	**Full Range**	**Variable**	**Coef.**	**p-value**
Nei's GD	Groups: CA & Baja	0.19	0.001	Model			
	TMax	0.07	0.016	GD ~ Groups + PrecipWQ	Groups: CA & Baja	3.52E-03	**0.001**
	PrecipWQ	0.21	0.001		PrecipWQ	2.60E-03	**0.003**
	PrecipDQ	0.15	0.005			**R^2 = 0.058**	**0.001**
**California Group**				**Southern California Group**			
Nei's GD	Euc. Dist.	0.42	0.001	Model	Mod. Habitat Cost	2.60E-08	0.001
	Mod. Habitat Cost	0.45	0.001	GD ~ Mod. Habitat Cost + TMax + PrecipWQ	TMax	1.22E-03	0.037
	TMax	0.14	0.006		PrecipWQ	2.97E-03	0.010
	TMin	0.18	0.007			**R^2 = 0.2302**	**0.001**
	PrecipS	0.28	0.001				
	PrecipWQ	0.36	0.001				
**Baja Group**				**Baja Group**			
Nei's GD	PrecipS	0.11	0.017	Model	PrecipS	1.13E-03	0.042
				GD ~ PrecipS		**R^2 = 0.012**	**0.042**

Table S3. Climate variable loadings from redundancy analysis for RDA axes 1–4. Scores represent the magnitude of each variable on the axis, the proportion of variation explained by each axis and the cumulative variation explained by each successive axis.

	**RDA1**	**RDA2**	**RDA3**	**RDA4**
TMax	0.58	-0.46	0.43	-0.49
TMin	0.55	-0.13	-0.07	0.16
PrecipS	-0.14	-0.16	-0.76	-0.25
PrecipWQ	-0.94	-0.05	-0.28	-0.16
PrecipDQ	-0.65	0.51	0.30	-0.47
Proportion explained	0.35	0.20	0.17	0.16
Cumulative proportion	0.35	0.55	0.72	0.87

Table S4: Full demographic modeling results from $\delta a \delta i$.

Definition	CAGN (log-L)	Parameters	Estimate	Definition	Parameter Convergence	Size change Description
3 Epoch: Multiple instantaneous size changes some time ago. Either a size increase in the second epoch or a bottleneck in the second epoch.	-550.64	nA	25,255	Ancient pop size	Yes	small to large to larger
		nu1a	110,519	Calculated from ratio of population size to ancient pop size		
		nu1b	393,980	Calculated from ratio of contemporary to ancient pop size		
		T1a	44,388	Time in the past at which instantaneous change happened		
		T1b	1,167	Time in the past at which instantaneous change happened		
Exponential Growth: Exponential growth beginning some time ago.	-1154.93	nA	25,605	Ancient pop size	Yes	small to large
		nu	178,277	Calculated from ratio of contemporary to ancient population size		
		T	64,360	Time in the past at which growth began (in units of 2*Na generations)		
2 Epoch: Instantaneous size change some time ago. This can be ancient-small to recent-large or ancient-large to recent-small.	-1577.46	nA	30,339	Ancient pop size	Yes	small to large
		nu	150,067	Calculated from ratio of contemporary to ancient population size		
		T	32,014	Time in the past at which size change happened (in units of 2*Na generations)		

Definition	CAGN (log-L)	Parameters	Estimate	Definition	Parameter Convergence	Size change Description
Growth Plus Bottleneck: Exponential growth beginning some time ago followed by a bottleneck.	-604.61	nA	7,126	Ancient pop size	No (nuB failed to converge)	large to larger to small
		nu1a	131,997	Calculated from ratio of population to ancience population size after exponential growth		
		nu1b	2,130,505*	Calculated from ratio of contemporary to ancient population size		
		T1a	171,095	Time in the past at which growth began		
		T1b	784	Time in the past at which instantaneous change happened		
standard neutral model	-13334.48	n/a	n/a		Yes	no change

Table S5: Full AMOVA results for tests with all loci and climate associated loci. Post-hoc pairwise Φ_{CT} tests follow. Bolded values for alpha ≤ 0.05 with B-Y corrections. Full dataset results below diagonal, climate associated loci above diagonal.

**Subspecies Hypothesis 1***						
	**All Loci**			**Climate Associated Loci**		
	**% variation**	**Φ**	**p-value**	**% variation**	**Φ**	**p-value**
**Among subspecies**	0.44	0.0044	0.001	0.98	0.0098	0.001
**Among populations within subspecies**	1.38	0.0139	0.001	1.85	0.0186	0.001
**Among samples within populations**	-1.99	-0.0203	NS	-1.70	-0.0175	NS
**Within samples**	100.17	-0.0017	NS	98.87	0.0113	NS

**Pairwise ΦCT***	***californica***	***atwoodi***	***pontilis***	***margaritae***	***abreviata***
californica	-	**0.0078**	**0.016**	**0.0164**	**0.0117**
atwoodi	**0.0033**	-	**0.0051**	**0.0041**	0.0013
pontilis	**0.0076**	0.0005	-	0.0026	**0.0044**
margaritae	**0.0091**	**0.0022**	0.0005	-	0.0011
abbreviata	**0.0053**	0.0001	-0.0003	-0.0005	-

**Subspecies Hypothesis 2****						
	**All Loci**			**Climate Associated Loci**		
	**% variation**	**Φ**	**p-value**	**% variation**	**Φ**	**p-value**
**Among subspecies**	0.40	0.0040	0.001	0.86	0.009	0.001
**Among populations within subspecies**	1.49	0.0149	0.001	2.10	0.0212	0.001
**Among samples within populations**	-1.99	-0.0203	NS	-1.70	-0.0175	NS
**Within samples**	100.10	-0.0010	NS	98.74	0.0126	NS

**Pairwise ΦCT****	***californica***	***margaritae***	***abreviata***
californica	-	**0.011**	**0.0064**
margaritae	**0.006**	-	0.0011
abbreviata	0.0025	-0.0014	-

* B-Y corrected p-value ≤ 0.018

**B-Y corrected p-value ≤ 0.027

Fig. S1.

Variability in five climatic variables across the study range.

1) Maximum temperature of the warmest month

2) Minimum temperature of the coldest month

3) Precipitation seasonality

4) Precipitation of the wettest quarter

5) Precipitation of the driest quarter

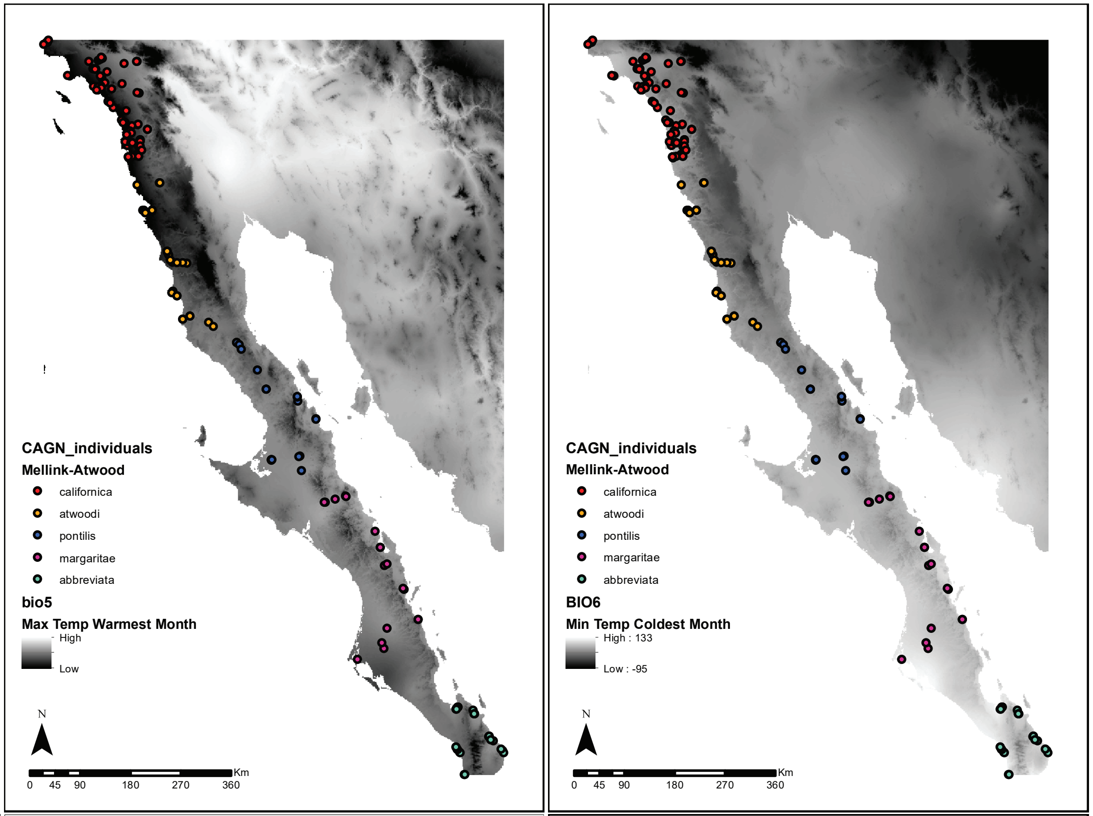

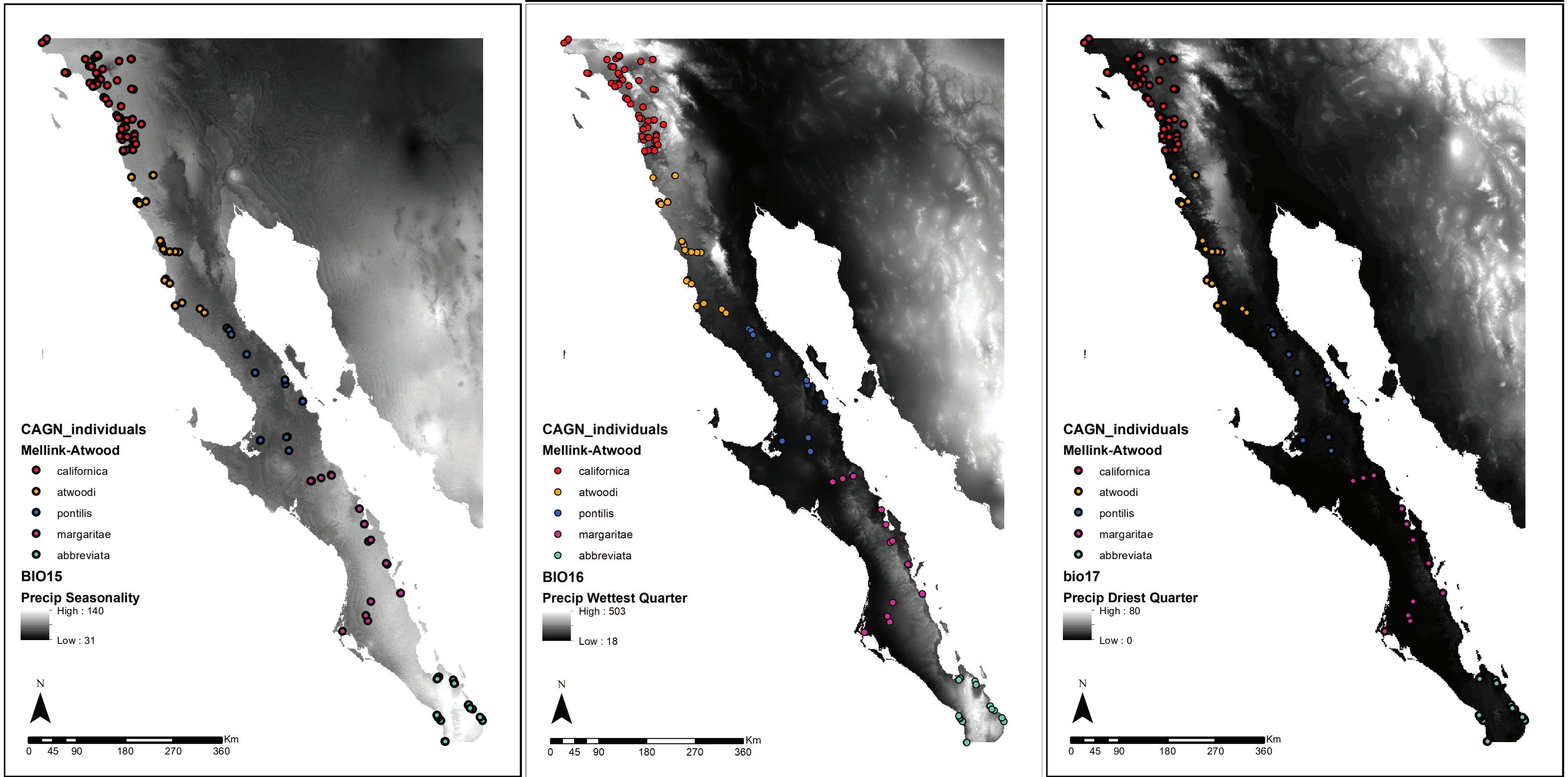

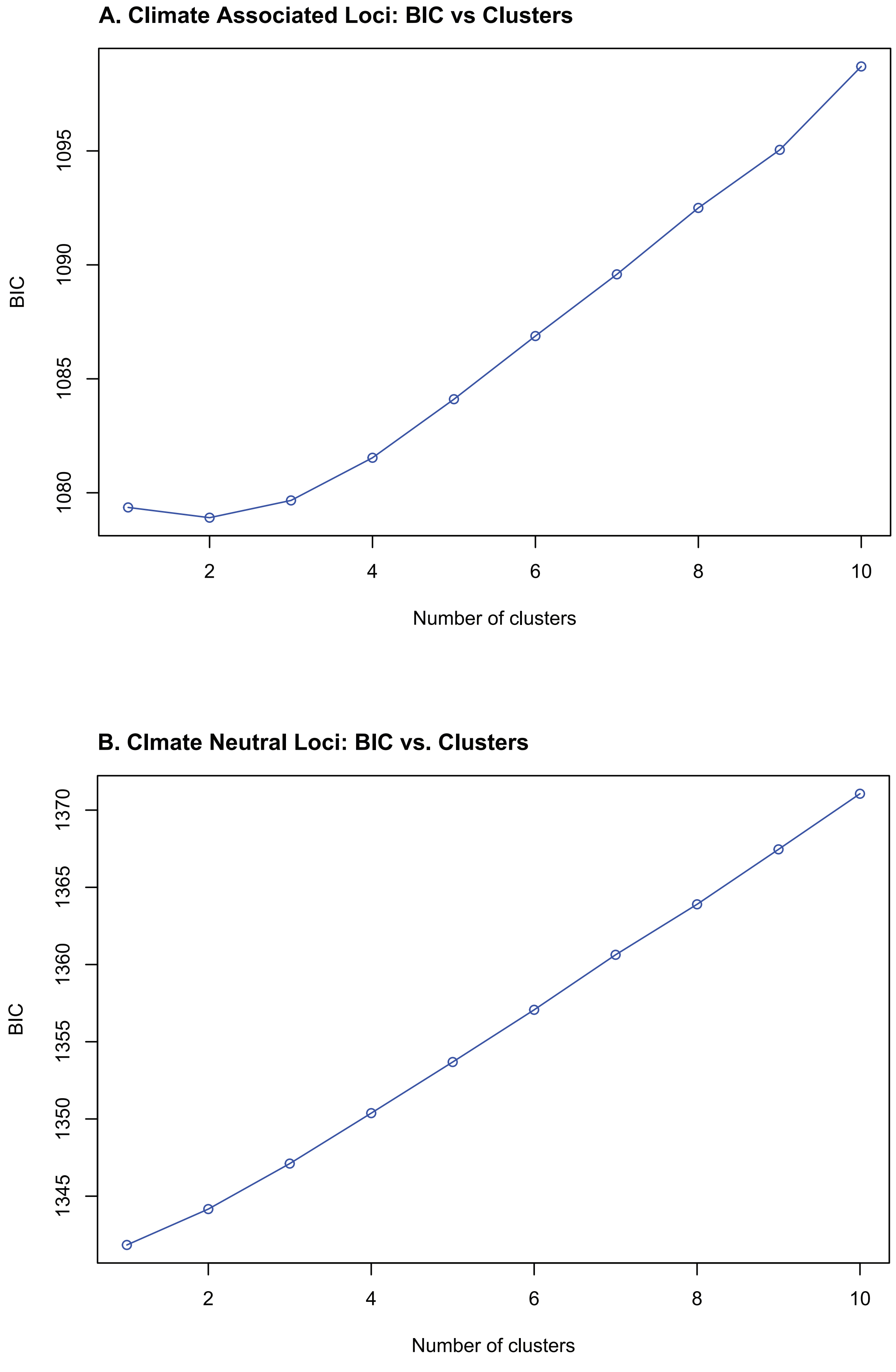

Figure S2: Plots of BIC scores by number of clusters for (A) climate outliers and (B) climate neutral loci.

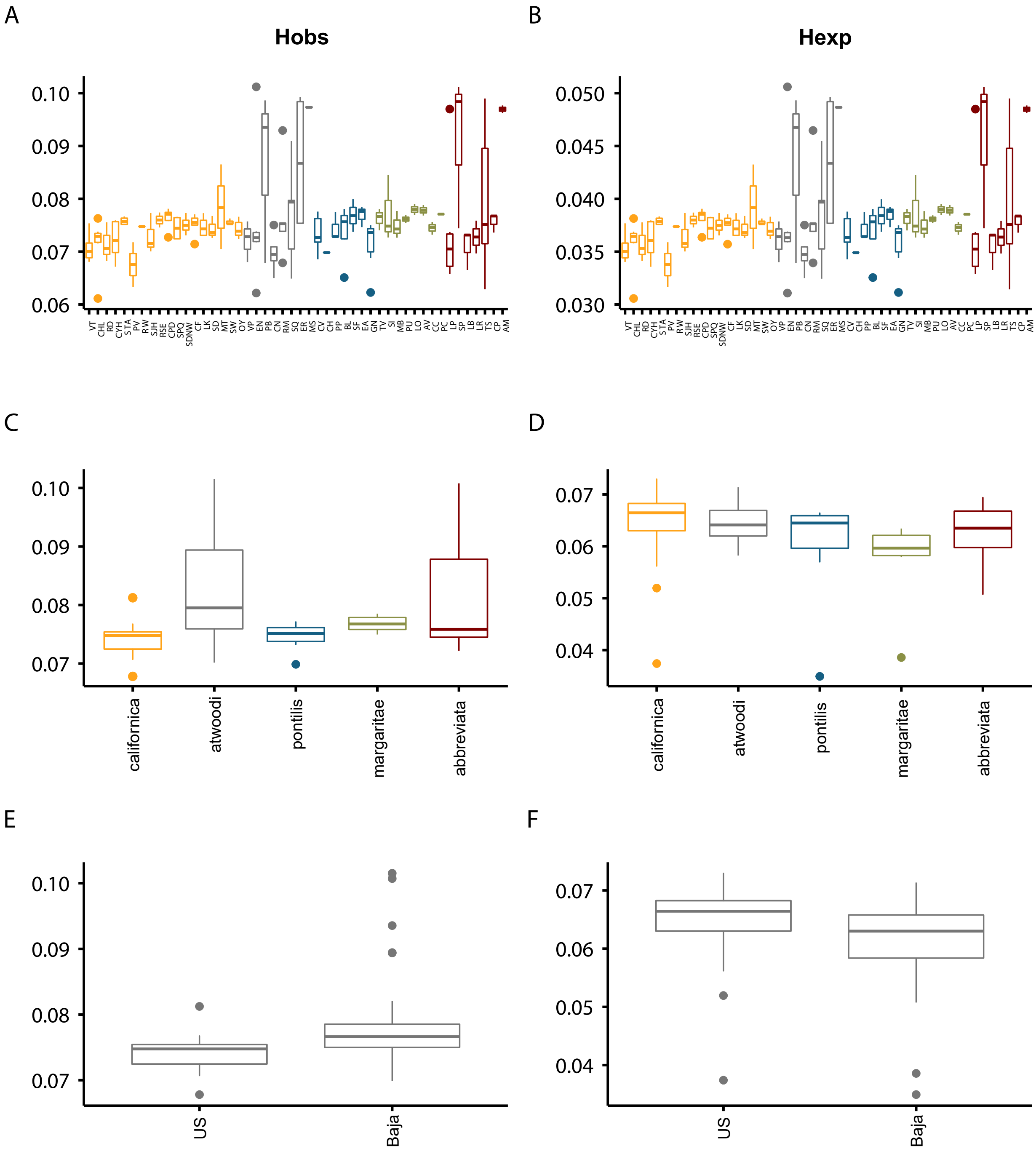

Figure S3: Diversity statistics. Boxplots of observed heterozygosity (Hobs) and expected heterozygosity (Hexp) for all sampled aggregations (A,B); for combined hypothesis 1 subspecies groupings (C,D) and combined for the southern California (U.S.) and Baja genetic groups.

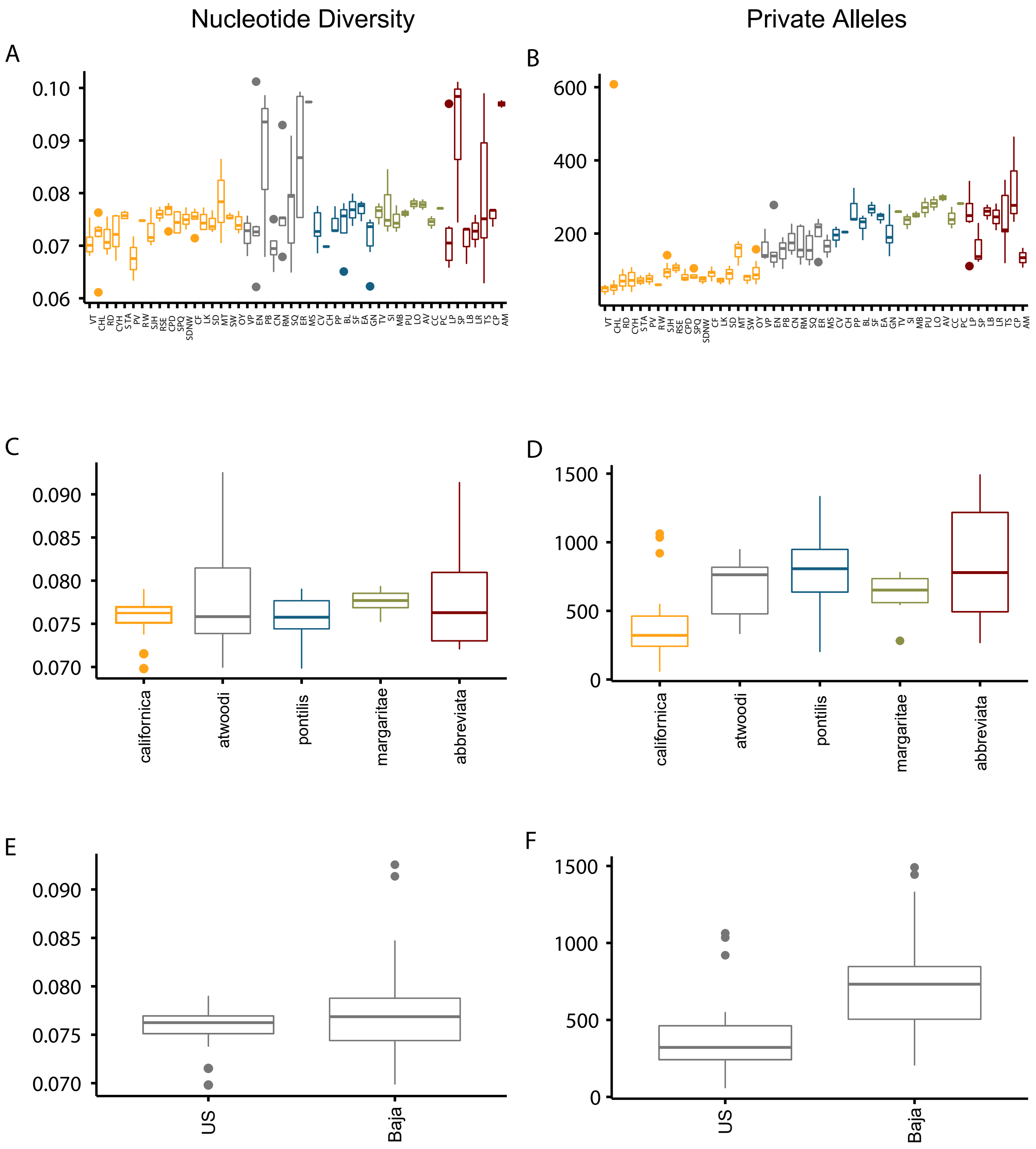

Figure S4: Diversity statistics. Boxplots of observed nucleotide diversity (pi) and total number of private alleles for all sampled aggregations (A,B); for combined hypothesis 1 subspecies (C,D) and combined for the southern California (U.S.) and Baja genetic groups (E,F).

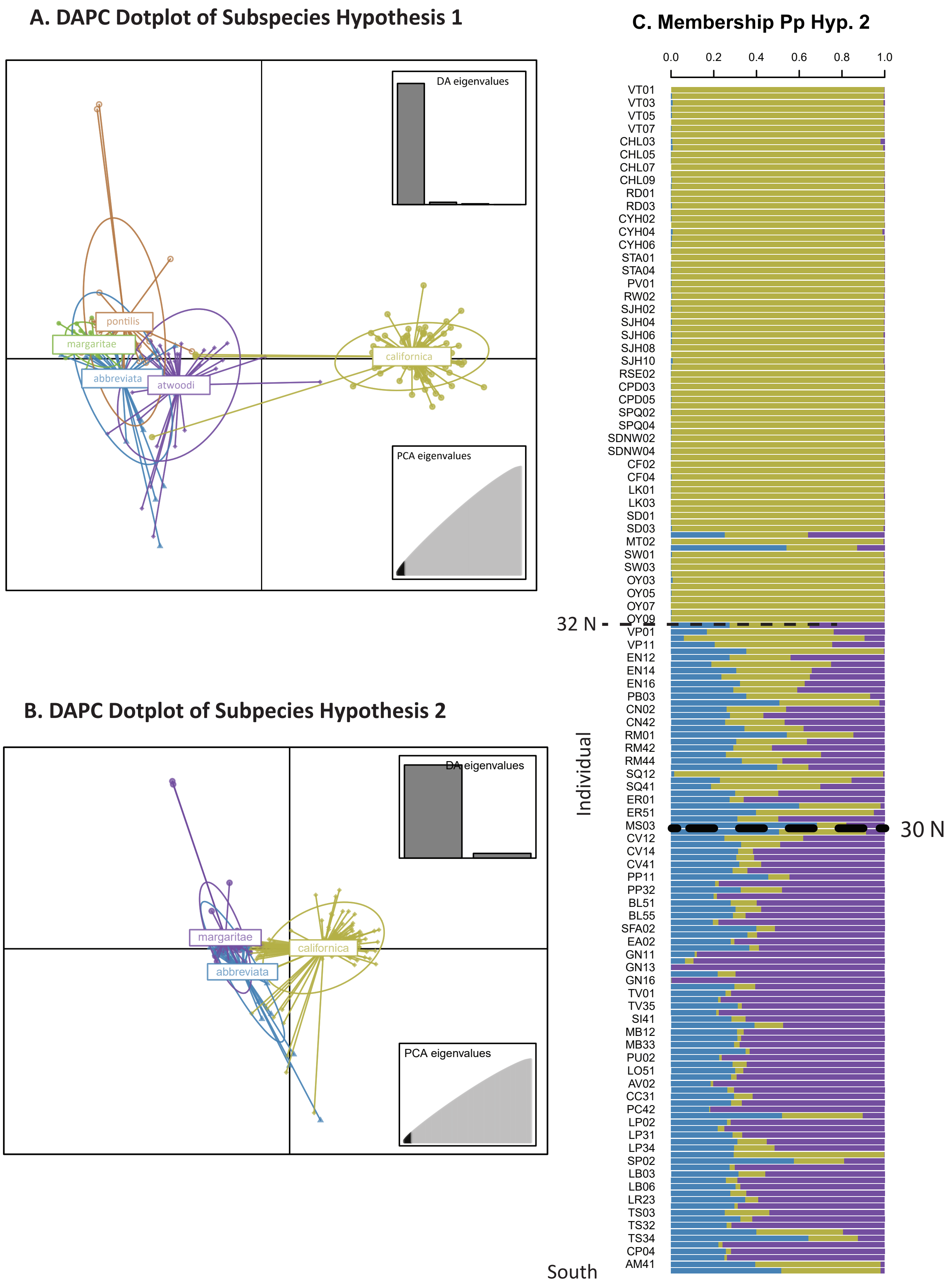

Figure S5: Discriminant Analysis of Principal Components (DAPC) results of subspecies hypotheses 1 and 2, optimized with 12 PCs. A. Subspecies hypothesis 1 Plot of DA axis 1 (horizontal) and 2 (vertical). B. Subspecies hypothesis 2 plot of DA axis 1 (horizontal) and 2 (vertical). C. Hypothesis 2 posterior probabilities of assignments arranged from North to South.
